# A framework to evaluate the thermal and energy performance of smart building systems in existing buildings: A case study on automated interior insulating window shades

**DOI:** 10.1016/j.mex.2025.103378

**Published:** 2025-05-19

**Authors:** Jongki Lee, Akram Syed Ali, Saman Haratian, Brent Stephens, Mohammad Heidarinejad

**Affiliations:** Department of Civil, Architectural, and Environmental Engineering, Illinois Institute of Technology, Chicago, IL 60616, United States

**Keywords:** Energy performance, Building science, Field measurement and validation, Heating ventilation and air conditioning systems, Energy efficiency measure, A methodological framework to evaluate the thermal and energy performance of interior insulating window shades in existing buildings

## Abstract

This paper presents a methodological framework for evaluating the thermal and energy performance of smart building systems in existing buildings, with a focus on automated interior insulating window shades as an energy efficiency retrofit measure. The methodology is demonstrated through a case study of a high-rise building in which different shade control strategies were assessed. This paper provides comprehensive descriptions of the (i) development and implementation of the study design, (ii) selection and deployment of measurement instruments, (iii) analysis of various shade control strategies to quantify thermal and energy performance for heating, cooling, and ventilation energy end-uses, and (iv) quantification of the uncertainty associated with the measurements and calculations. This manuscript provides detailed, step-by-step and in-depth guidance to conduct such an evaluation. Overall, this paper:

• Highlights the benefits, challenges, and limitations in conducting long-term measurements that capture realistic temporal, seasonal, and operational patterns in an occupied existing building.
• Provides practical considerations for such measurements and analysis approaches, upon which future studies can build.
• Emphasizes the importance of ensuring that the study design and measurements do not interfere with the building’s existing operations.

## Graphical abstract

**Figure:**
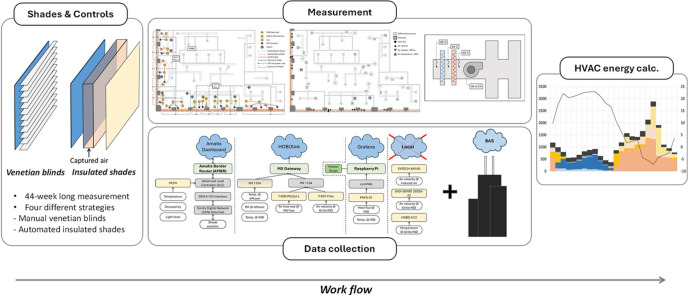

Specifications table

**Table:** 

Subject area:	Engineering
More specific subject area:	Measurement and data analysis in an existing building to assess energy performance
Name of your method:	A methodological framework to evaluate the thermal and energy performance of interior insulating window shades in existing buildings
Name and reference of original method:	Jongki Lee, Akram Syed Ali, Afshin Farmarzi, Urwa Irfan, Christopher Riley, Brent Stephens, Mohammad Heidarinejad, Assessing the long-term energy performance of automated interior insulating window shades in a high-rise commercial building, Applied Energy, Volume 378, Part B, 2025, 124,797, ISSN 0306-2619, https://doi.org/10.1016/j.apenergy.2024.124797.
Resource availability:	All the instruments and their datasheets are cited in the text.

## Background

Window shading systems in buildings offer benefits for energy performance, including reducing heating and cooling loads by blocking solar radiation and minimizing heat transfer between the indoors and outdoors [1,2]. Window shades can also shift peak heating and cooling demands, which can impact the timing and magnitude of energy use and thus affect a building’s participation in demand response programs [3]. According to a recent study [4], windows can account for 37 %−50 % of energy dissipation among building envelope components, while the building envelope contributes about 50 % to heating and cooling energy loads overall. Therefore, the installation of a window shading system is a promising energy efficiency measure (EEM) in buildings, especially in older existing buildings where other building envelope EEMs are limited.

There are various types of window shading systems, differing based on their placement (e.g., exterior, intermediate and interior), composition or material (e.g., thin metal slate, mortar, cloth), type (e.g., overhangs, louvers, vertical fin, vertical blinds, roller shades), and operation (e.g., static and dynamic) [2,5,6]. Window shading systems are typically used to (1) improve visual comfort, (2) optimize daylighting, and (3) reduce heating, cooling, and lighting energy end-uses. Control variables for adjusting the shades’ position may vary depending on the objectives of study or application. In visual comfort, lighting, or daylighting studies, several common parameters that are commonly used to optimize the target conditions include daylight glare index (DGI) [7], discomfort glare probability (DGP) [[8], [9], [10], [11]], daylight [9], vertical and horizontal illuminance [8,12,13], work plane illuminances [9], and global horizontal illuminance (GHI) [14]. Existing studies have investigated these aspects, such as reducing heating, cooling, and lighting energy end-uses by using different types and shapes of shading devices [15,16] and utilizing various settings and control sequences [17]. Here we focus on HVAC energy savings.

Most of these studies have been conducted using numerical modeling and simulations [18] or with measurements in laboratory settings [19], under controlled environments such as test houses [[20], [21], [22]], or for a limited duration during field studies [23]. Two technical reports published by Pacific Northwest National Laboratory (PNNL) evaluating HVAC energy savings associated with a specific shading system similar to the design of this study [24,25]. Without occupants, the control experiment is for a baseline strategy and the case experiment is for the shade. The researchers implemented five different control strategies including an optimal mode, a dynamic mode, a demand response mode with a mean temperature throughout the home, and a typical mode assuming occupant behavior [25]. These studies firmly divided heating and cooling periods to determine each season’s energy savings. In terms of duration for each scenario, including variation of settings and control sequences, the demand response study in the cooling season was conducted for 25 days while most of the others lasted for approximately 8 days. Informed by this study, we tailored our experimental design to capture longer periods to understand the performance of shades over different seasons and actual occupant interactions.

## Method details

Capturing the effects of shade systems under realistic conditions in real buildings necessitates longer-term measurements that capture interactions of occupants with the window shades rather than using models to set the shades’ positions (e.g., up or down) based on pre-defined schedules. While exterior shades are typically designed and installed during the building’s design phase, interior shades are often installed as a retrofit measure, which can present challenges such as limited spaces for installation of the interior shades and their controllers. In conducting an investigation of the energy performance of interior insulating shades in an existing high-rise building, it became apparent that there is limited information on appropriate research methodologies, instrumentation, analysis approaches, and even integration of shading systems with automated controls and data acquisitions. Yet, these details are important for introducing, monitoring, and evaluating smart building concepts to existing buildings. Therefore, this study aims to provide details on a methodological framework to demonstrate how the energy performance associated with interior shade solutions could be evaluated in a real building under realistic conditions. Through this study, we explicitly articulate not only details in design and instrumentation of the case study but also lessons learned, best practices, and limitations in each subject to inform future work. All figures and tables and information here were taken from the real measurement and analysis.

This study presents an experimental design for the long-term performance evaluation of automated interior insulating roller window shades in an actual occupied office building conducted over a 44-week long data collection period (from May 03, 2021 to March 06, 2022) that the results were demonstrated in this main paper [26]. This study also provides details on the field installation and instrumentation required for the performance evaluation. The concept of interior insulating shades refers to benefiting from the air gap between the window and the shade to alter the overall R-value of the glazing system [27]. This concept allows blocking heat transfer between the glazing and shade cloth while the shades are attached to the window frame. This study expands the applicability of insulating interior shades by implementing different control strategies and sequences of operations (SOOs) to modulate the shade positions. Overall, this study will be valuable for future studies aiming to (1) design and conduct a long-term evaluations of shading systems (or similar building systems) with various control strategies, (2) calculate potential heating and cooling energy end-uses when sub-metering the main electricity panel or electric circuits is not feasible, necessitating additional instrumentation to indirectly calculate energy consumption, and (3) quantify propagated uncertainty in calculated energy consumption using the installed instrumentation. Such measurements and lessons learned may also be extended to inform assessment of the thermal and energy performance of other building components and systems.

## Case study building

**Fig. 1:**
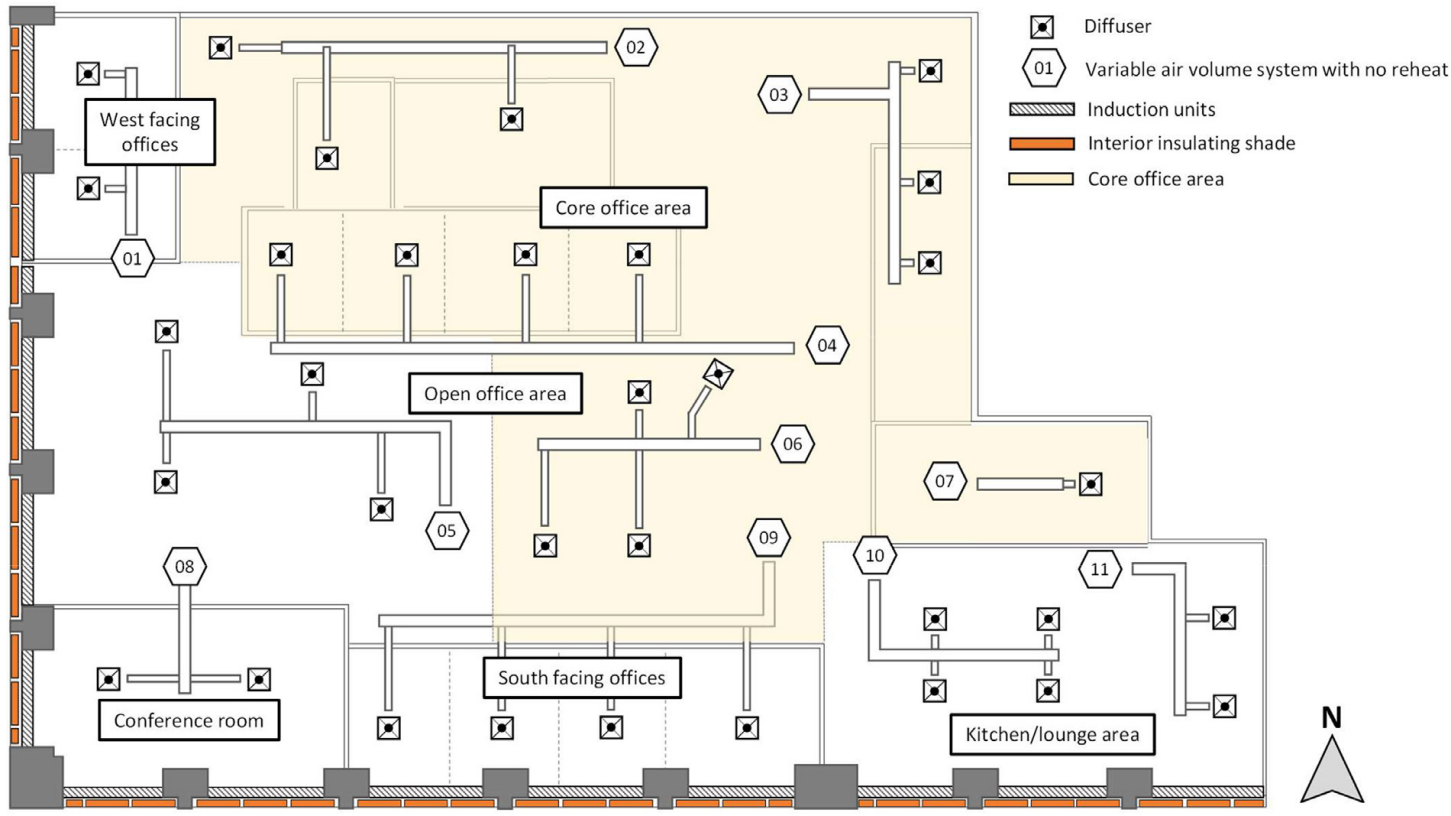
The schematic of the case study area illustrating the layout of the mechanical systems, shades, and spaces.

The case study is a mixed used office area in a high-rise commercial building in Chicago, Illinois, USA. The case study area faces both the south and west orientations and has a floor area of 672 m^2^ (7233 ft^2^). This study installed 39 motorized insulating interior shades to cover all the windows of the space. Fig. 1 shows the schematic of the case study area.

The south-facing orientation includes 24 windows, with a combined width of 33.2 m (109.0 ft) and a height of 3.0 m (9.9 ft), resulting in an exterior window surface area of 100.2 m^2^ (1078.9 ft^2^). The west-facing orientation includes 15 windows, with a combined width of 20.6 m (67.7 ft) and a height of 3.0 m (9.9 ft), resulting in an exterior window surface area of 62.2 m^2^ (669.8 ft^2^). There are three different sizes of windows: (i) 144.8 cm (57 inch), (ii) 132.1 cm (52 inch), and (iii) 55.9 cm (22 inch), with 12, 22, and 5 windows of each size, respectively. In Fig. 1, the white area indicates the perimeter zone that is directly impacted by the shades, and the yellow area represents the core office zone that is less directly affected by the interior insulation shades.

For this study, over 190 data variables were collected from either the monitoring instruments deployed by the research team or the building automation system (BAS). A detailed understanding of the existing HVAC systems was important for assessing heating and cooling energy consumption patterns in the case study area. The space was equipted by two main HVAC systems to regulate the indoor temperature and provide ventilation: (1) a perimeter induction unit system located under each window which delivers and recirculates air through zone-level heat exchangers, and (2) a variable air volume (VAV) air distribution system located above the drop ceiling, which delivers air to the space through ceiling diffusers. The VAV system had no reheat. The VAV system was installed in each space (thermal zone) and controlled by the wall mounted thermostats. The induction units, sometimes referred to IND in this study, were located in the perimeter zone and their operation was modulated by both the BAS and slightly adjusted by the thermostat dials on each induction unit. They were continuously in operation based on the BAS schedule while the VAV system turns on and off based on the wall mounted thermostats. Fig. 1 shows these two systems.

## Development of the study design

This study design section details the development of methods to assess the thermal performance of interior insulating shades in a case-control study. Specifically, the study aimed to assess building energy consumption patterns with the focus only on heating and cooling savings. Therefore, understanding the performance of HVAC systems and associated parameters was required. To measure these parameters, either sensors can be installed, or the needed data can be obtained from BAS, or a combination of both; this study relied on both approaches. The sensor selection required measuring energy performance including the rate of sensible heat transfer in both the central HVAC system, which is located inside of the air handling units (AHUs), and the zone level systems, which include induction units, VAV boxes, and diffusers serving the occupied space. The collected data for this study therefore include:

(1) Air velocity inside the induction unit system to assess the air flow rate being delivered to the space
(2) Differential pressure reading before and after the VAV boxes to assess the air flow rate of each VAV box serving the space
(3) Temperature values either inside of the induction unit system or before and after of the air delivery system serving the space
(4) Temperature values delivered by the ceiling diffusers from the VAV boxes
(5) Temperature values before and after the heating and cooling coils in the AHUs and/or damper control devices to calculate temperature differences to calculate the heat transfer rate
(6) Heat flux sensors on the windows, shades, and induction unit to measure the temperature and heat flux on different surfaces
(7) An integrated occupancy and lighting density sensor to measure the room occupancy and light density level
(8) The shade position from the controller to understand how often the shades are in the binary mode of up or down.

**Fig. 2:**
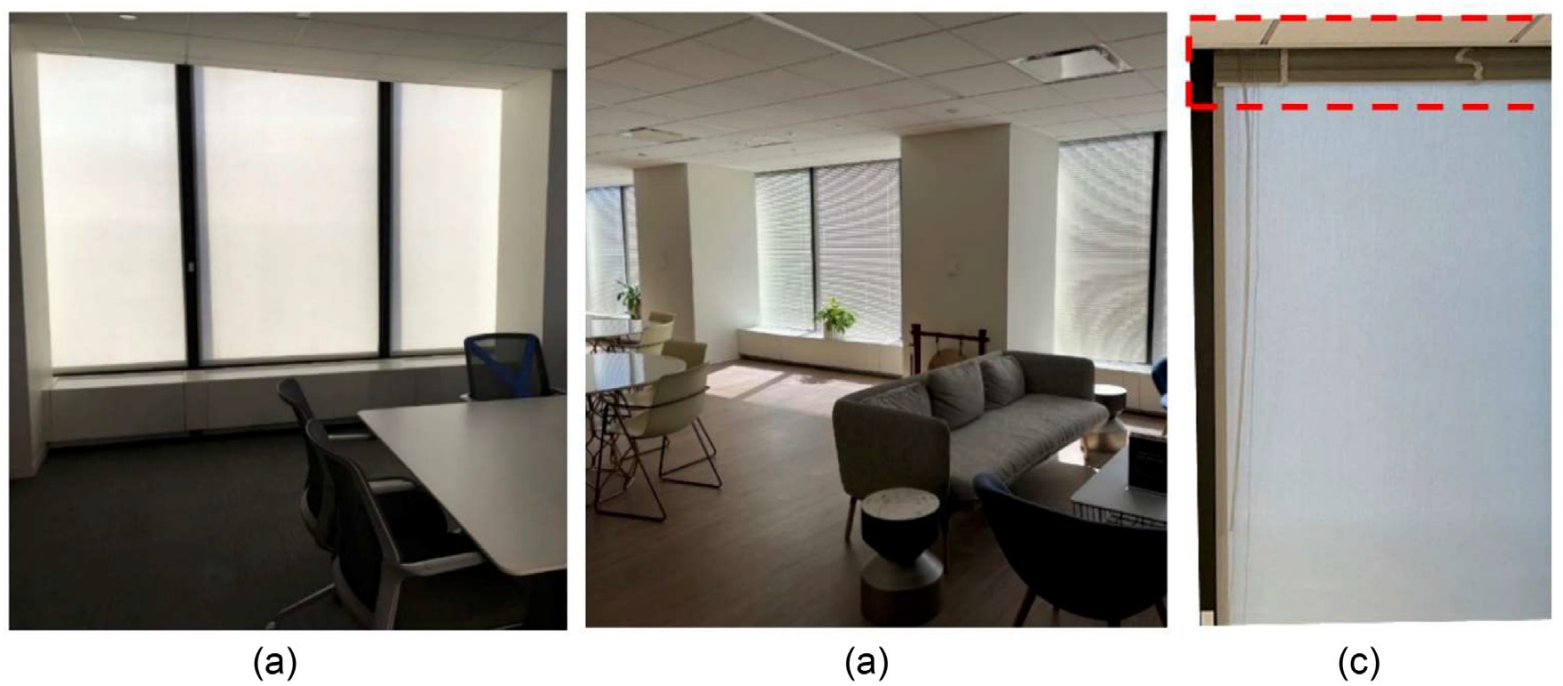
Demonstration of the interior insulating shades and mini-blinds: (a) when the motorized interior shades are in operation, (b) when mini-blinds are in operation for the Baseline strategy, and (c) when the motorized interior shades are in operation and the mini-blinds are all the way up (a close view).

To evaluate the performance of the interior insulating shades and understand the impacts of automating them with different control sequences, this study developed and applied four different control strategies. Out of the four strategies, three of them are based on the interior motorized insulating shades (cases) and one utilizes mini-blinds as a baseline (control). The goal was to compare the performance of motorized insulating shades with respect to mini-blinds. Both the motorized insulating interior shades and the mini-blinds were installed in parallel to each other as Fig. 2 shows. Before their installation, the space had an older version of the shades. First, the new motorized insulating shades were installed for all the 39 windows. Then, 29 mini-blinds were installed in representative spaces: (1) the kitchen/lounge, (2) two south-facing offices, (3) the main conference room, (4) the open space area, and (5) one westfacing office. These spaces were selected to account for unique spaces and also minimize the cost of the project. The selected spaces all include the thermostat for controlling the VAV boxes, and the impact of not covering the entire windows with the mini-blinds were minimized.

The Baseline strategy included the installation of mini-blinds, as they are typically used as existing window covering devices in this building and a significant number of existing buildings. The motorized shade strategies included (i) manual operation (called Manual), in which occupants set the shade position to only up and down based on their preference, (ii) a scheduled-based (called On-schedule) strategy, similar to temperature thermostats and lighting schedules, which uses pre-defined and optimized schedules for building energy consumption based on the season and time of the day [28], and (iii) a Dynamic (or Smart) strategy that aims to optimize the operation and position of the shades based on indoor and outdoor conditions. The logic behind the development of these three motorized insulating shade control strategies was to compare the energy consumption to Baseline and also learn the best SOOs in each season.

**Fig. 3:**
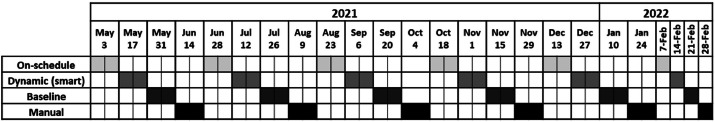
The order of control strategies based on the two-week measurement.

Measurements were conducted over 10 months (May 03, 2021 to March 06, 2022). Each control strategy was deployed for two consecutive weeks, rotating to the next strategy for the next two weeks, and so on. This alternating pattern repeated until each strategy was deployed for a total of 8 weeks (four repetitions of two weeks for each strategy), which allowed for each strategy to be deployed during each season. Fig. 3 shows the order of strategies began with 2 weeks On-schedule (started May 3, 2021), followed by 2 weeks each of Dynamic, Baseline, and Manual, and then repeated three more times. In the last cycle of measurements (started February 14, 2022), each strategy was deployed for only one week (although in the same order) to capture more extreme winter weather at higher resolution.

Throughout the study, occupants had the opportunity to interact with the motorized shades and change their position in all the shade strategies. The Manual control strategy allowed the study to investigate the interaction between occupants working in the office area and the shades to understand how they prefer to position the shades. It is noted that during the automated control strategies (i.e., On-schedule and Dynamic), occupants were still able to override the position of shades when they wanted to exert control. When the occupancy sensor detects no occupancy in the office for 15 consecutive minutes, the shades follow the automated schedule for both the On-Schedule and Dynamic strategies. Based on the results from the interaction between occupants and the shades, this study learned how the strategies could be optimized for future study. The study also conducted a short survey after the experimental period to ask about their preferences for the shades. Thus, in future studies, it is important to explore in more depth how human perception could affect performance of smart building systems (i.e., a shading system) and how that could impact energy consumption and potentially interrupt their preference, such as why and when occupants control the shades.

## Design and installation of interior insulating shades and sensors

The concepts of interior insulating Parata shades used in this study had been patented [27,29]. Thus, this study was designed to evaluate the performance of these interior shades with different control strategies operated and tested as an automated interior insulating shades system in an actual building application. Therefore, there was a need to carefully develop several aspects of the shade and study implementation: (i) how to set the shade position, (ii) how to collect the shade’s position, (iii) how to enable controlling the shade position using both the application programming interface (API) and the graphical user interface (GUI) in a dashboard, and (iv) how to transmit the real-time data to the cloud. The concept of shade control zoning is also used to group the shades in one space. The kitchen space in Fig. 1 has nine shades and were grouped into one shade control zone, and other shade control zones group into a number of shades in their space. Each shade control zone is operated by one remote switch that sets the shade position by occupants. In addition, the system is programmable, allowing to set different schedules for a certain period. The other feature that was considered was to record the position of the shades and other information (i.e., level of light (%) and occupied (binary)).

**Fig. 4:**
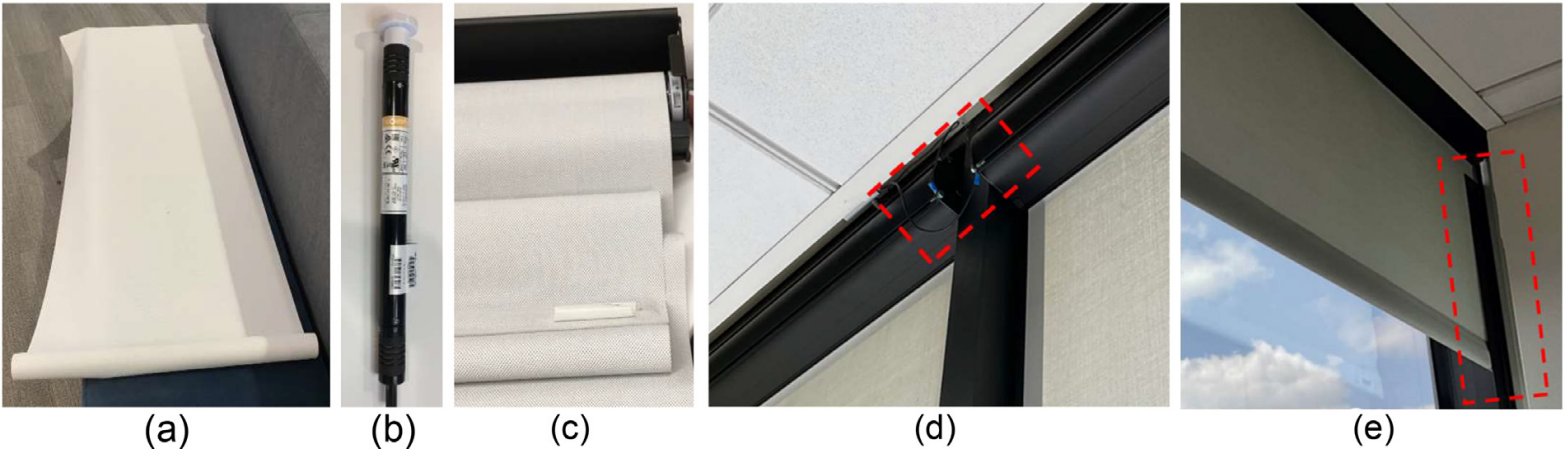
The physical portions of the installed shade system: (a) the shade cloth, (b) the motor (actuator), (c) the shade cloth and the motor in the casing, (d) the static removal and grounding solution, and (e) the l-guide extension.

Fig. 4 summarizes the physical components of the motorized shading system used in this study. The shade is Phifer Oyster/Pearl Gray SheerWeave 2500 1 % [30], as shown in Fig. 4(a). Fig. 4(b) shows the motor, which is a Somfy Sonesse ULTRA 30, acting as the actuator [31]. The two components are assembled in a casing shown in Fig. 4(c). Fig. 4(d) and (e) show how we addressed potential problems after installation. In spaces with floor carpets, cloths, and other elements that could build up static charge, static removal was important to avoid damaging the electronics [32]. As shown in the red dashed box in Fig. 4( d), the shade system was grounded to avoid any static charge build up and damage to the motor and the controller. Also, it is important to install the controller inside of the system and make it resistant to static build up, especially for the wintertime. The red dashed box in Fig. 4(e) shows the l-guide that was installed before the beginning of field experiment to ensure the shades follow the mullions. Since some of the west-facing window mullions were wider than the shade cloth, they were not able to guide the shade cloth. The l-guides allowed the shade cloth to move up and down properly. Without the l-guide, these shades were not able to move up and down as expected and due to unbalanced roll up, they ultimately led to the shade cloth getting stuck in the casing. The l-guides, as a standard solution, were adhered to the mullion to provide additional vertical face area. Size of the l-guide is 5 cm x 5 cm (2″x2”) or it could be increased if more support was needed. Finally, the upper and lower height of the shades as shown in Fig. 2(a) were set by the Somfy RS485 setting tool to ensure the air gap between the window and shades were fully covered and limit the amount of convection and solar radiation to the space when the shades were fully down [33].

**Fig. 5:**
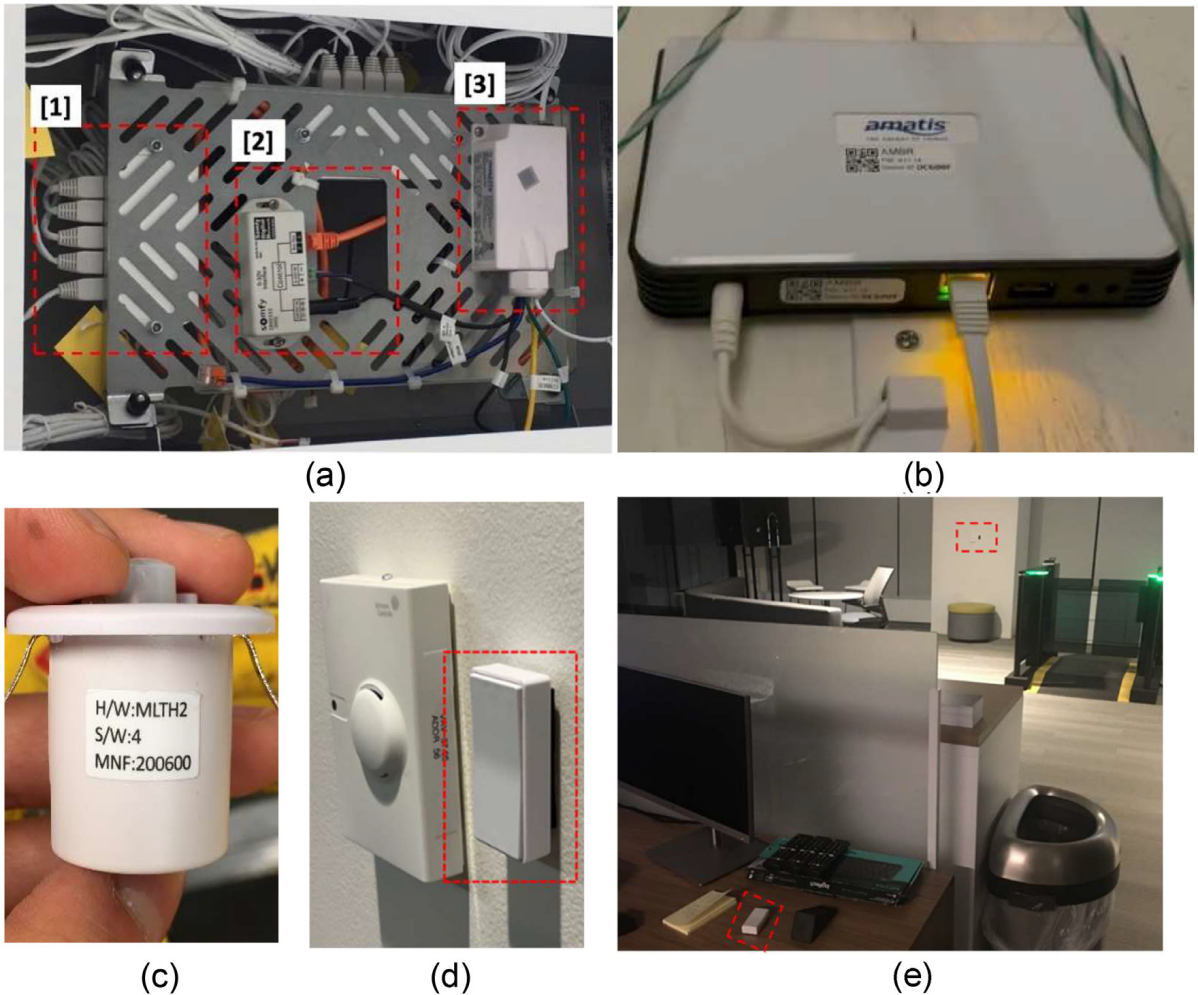
The control portions of the installed window shade system: (a) control panel, (b) communication router, (c) MLTH sensor, (d) shade switch next to a room thermostat, and (e) the removal of the control switch from the wall, so the occupant can easily control the shade.

Fig. 5 shows the control portions of the shading system, which is integrated with the physical portions shown in Fig. 4. The shade system is connected to a data hub, Somfy Digital Network (SDN) Data Hub, which receives and transfers the signal with up to 5 channels with RS485 cable (RJ12) [34] shown in Fig. 5(a), Part [1]. Part [1] connected to a SDN 0–10 V Interface with a RS485 cable [35] as shown in Fig. 5(a), [2]. Part [2] operates the shade to fully close and open with input voltage range 0–10 V. The Advanced Load Controller (ALC) shown in Fig. 5(a), Part [3], is able to output voltage from 0 V to 10 V for a dimming control and to communicate with an Amatis Border Router (AMBR) to transmit the real-time data to the cloud system [36]. Fig. 5(b) shows the AMBR. This device receives the signal (data) from the ALC through 6LoWireless and transmits to Amatis Dashboard/APP via LAN or BMS [37,38]. In addition, the ALC has a port for a compatible sensor called Motion, Light, Temperature, and Humidity (MLTH), shown in Fig. 5(c) [39]. To communicate to ALC, it uses 6LoWireless communication protocol allowing data and command crossover [40]. In this study, occupants use a control switch to adjust shade position as shown in Fig. 5(d) [41]. The occupants have a binary control option to either set the shade as up or down. The shade position signal communicates with ALC by using 6LoWireless communication protocol. During the experiment, some occupants would like to control the shades more often, especially during the On-Schedule or Dynamic strategies when the occupancy sensors adjust the shade position when the vacancy status is detected. These occupants would like to override the system and benefit from the visual view of the environment. Fig. 5(e) illustrates when one of the control switches for the shades were moved daily in the open office area and the occupant continuously modified the shade position.

**Fig. 6:**
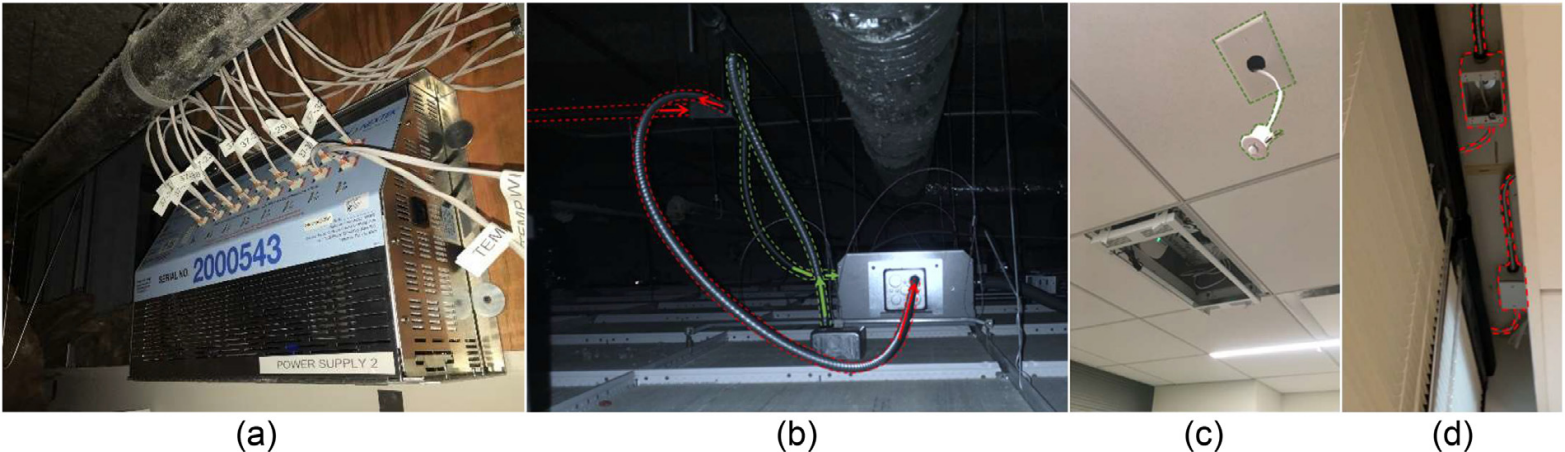
The low-voltage connections for the controllers and the shades: (a) power supply, (b) conduits for the low voltage, (c) MLTH sensor and the control box in the ceiling, and (d) electrical junction box for the shades to controllers.

The motor and control devices operate based on low voltage power. Consequently, power supplies and low voltage wires were installed throughout the test study area above the drop ceiling as shown in Fig. 6. In addition, the low voltage power was run in electrical conduits to meet Chicago building code requirements. Fig. 6(a) shows the power supply that converts the 110 VAC (Volts Alternating Current) to 24 VDC (Volts Direct Current) for the controller and the shades. Fig. 6(b) shows the control box in the ceiling (the box is also shown in Fig. 5(a)). The box is fed with the low voltage from the power supply and provides power to the motor and the sensor as Fig. 6(c) and (d) shows. The green dashed line in Fig. 6(b) and (c) shows the low-voltage conduit extended to the MLTH sensor to also receive the measurement data. The red dashed line in Fig. 6(b) and (d) shows the conduit from the control box to the motor. With the optimization of the system, future studies could utilize the Power over Ethernet (PoE) instead of running the low-voltage line and power supply.

**Fig. 7:**
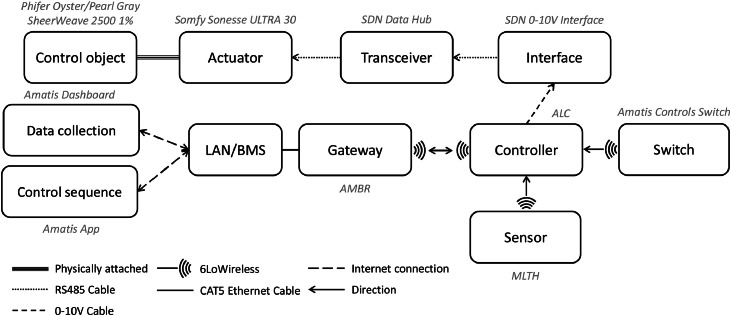
The configuration of automated shade systems physical and control components together.

Fig. 7 summarizes the configuration of the automated shade system and their communication and control components. Once the ALC controller receives the signal to operate the shades, it sends the data to the actuator through the interface and the transceiver. The signal transmits either from the Amatis App or the control switch, and the ALC controller sends the operating level and data from the MLTH sensor to the server (Amatis Dashboard). There are two methods to download the data either through: (i) a python script, which allows for downloading the raw data or (ii) through the Amatis Dashboard, which allows downloading the processed data [38,42]. Furthermore, there are two methods to configure the control strategies: (1) using the Amatis Controls to set the shade position for each day, week, and time of the day and (2) using another python script that allow adding extra outside features such as the outdoor air temperature for the Dynamic strategy.

## Quantification of heating and cooling energy consumption as target objectives

This section describes how heating and cooling energy consumption were quantified as the target objectives. Since the building is all electric, for the calculation of heating and cooling, the best approach would be to directly measure AHUs, boilers, and chillers’ electricity consumption in their dedicated electric circuit or panels connected to these HVAC components to sub-meter the panels. This approach has been used before in the literature for a testbed [43], operation of fans [44], and operation of plug loads [45]. Also, existing studies evaluated energy performance of shading devices using an energy logger [46] and collecting the energy data from panels from whole building level to appliances and lighting level panel [24]. However, given the complexity of the systems delivering heating, cooling, and ventilation to this space, a submetering approach was not practical. First, the size of the AHU is very large, while the test space on floor 37 is served by a relatively small portion of the airflow, and in complex ways. The total air flow rate coming from four AHUs supplying air to the VAVs on floors 4 through 49 is 1168,920 m^3^/h. In addition to the four AHUs supplying air to the VAVs, there are two additional AHUs that supply air to the induction units. While the building has gone through retrofit of HVAC systems and it is equipped with a BAS, the energy consumption is monitored primarily at the building level, not the space level. Therefore, this study had to design a custom approach to monitor variables needed to calculate heating and cooling energy consumption patterns for the test space.

To estimate the HVAC energy consumption serving the test space, including the VAV and induction unit systems, measurements focused on assessing sensible heat transfer delivered to the space. With the low occupancy rate of the space and non-existence of any humidifiers or dehumidifiers in the main AHUs, this assumption is reasonably valid. Eq. (1) shows the governing equation to calculate the sensible heating and cooling energy delivered to the space:

(1) $$\dot{Q}=\dot{V}\times \rho \times C_{p}\times \Delta T_{1-2}$$

where, $\dot{Q}$ is the HVAC energy delivered at the AHU level (kW), $\dot{V}$ is volumetric air flow rate (m^3^/s) from the outlets of system, ρ is air density (1.225 kg/m^3^ at sea level and 15 °C), $C_{p}$ is the specific heat capacity of air (1.006 kJ/kgK), and $\Delta T_{1-2}$ is a temperature difference across a coil. To use this equation one should measure (or collect) the volumetric air flow rate and two temperature points (e.g., before and after heating or cooling coil) over time.

The actual HVAC energy consumption depends on the fuel type and can be calculating knowing the efficiency of the system:

(2) $${\dot{Q}}_{Actual\;consumption}=\frac{{\dot{Q}}_{calculated}}{COP\left(or\;\eta_{efficiency}\right)}$$

where, ${\dot{Q}}_{Actural\;consumption}$ is the calculated energy consumption by HVAC system (VAV or Induction units) (kW), ${\dot{Q}}_{Calculated}$ is the $\dot{Q}$ in Eq. (1), and COP indicates coefficient of performance. The current study used a COP of 4 for the cooling coil based on 10 s interval data analysis on chiller COP [47,48] and used an efficiency ($\eta_{efficiency}$) of 1 for an electric heater. For a more conservative calculation, 2.4 could be applied for COP, which is an average value of existing buildings in the U.S. obtained from ComStock [49], or an efficiency of a gas fired boiler ($\eta_{efficiency}$) could be assumed to be 0.77 if applicable, which is an average value of existing buildings in ComStock [49], or 0.8–0.9 as typical recommended assumption [50]. However, the best practice is to use as many of the actual specifications on the building and its systems under study, to the extent that they can be known.

This analysis only considered the temperature difference before and after coils. However, we observed some unreasonable, illogical temperature differences (e.g., heat addition during the peak summer or heat reduction during the peak winter). Furthermore, there are shoulder seasons, spring and fall, that use less heating and cooling energy than other seasons and/or use mixed heating and cooling energy together, with no primary contribution to the heating or cooling energy consumption. This study used an indicator to determine whether the AHUs are providing heating or cooling with its certain intent. From the BAS, one of the recorded values clearly designates intent of HVAC operation; i.e., “summer” or “winter”. Thus, when the indicator says “summer”, then the AHUs provide cooling, not heating, and the analysis counts only negative values of temperature difference ($T_{before\;coil}>T_{after\;coil}$). A “winter” case has the same logic ($T_{before\;coil}<T_{after\;coil}$). The best practice for analyzing sensible heat energy consumption in HVAC systems is to use a proper indicator representing the HVAC system’s operating purpose, which may require additional measurements rather than relying on a BAS for data collection, even if redundant.

For most of the data, the measurements were collected at 1- and- 5 min intervals. However, data from the BAS were collected at 10- and 30-min time intervals. To address this issue, the acquired data from either measuring instruments or BAS were regarded as having a 1-minute time interval. In other words, 5-, 10- and 30-min intervals were regarded as 1-min data (i.e., larger time steps than 1-minute is constant during its interval).

### Variable air volume (VAV) system

**Fig. 8:**
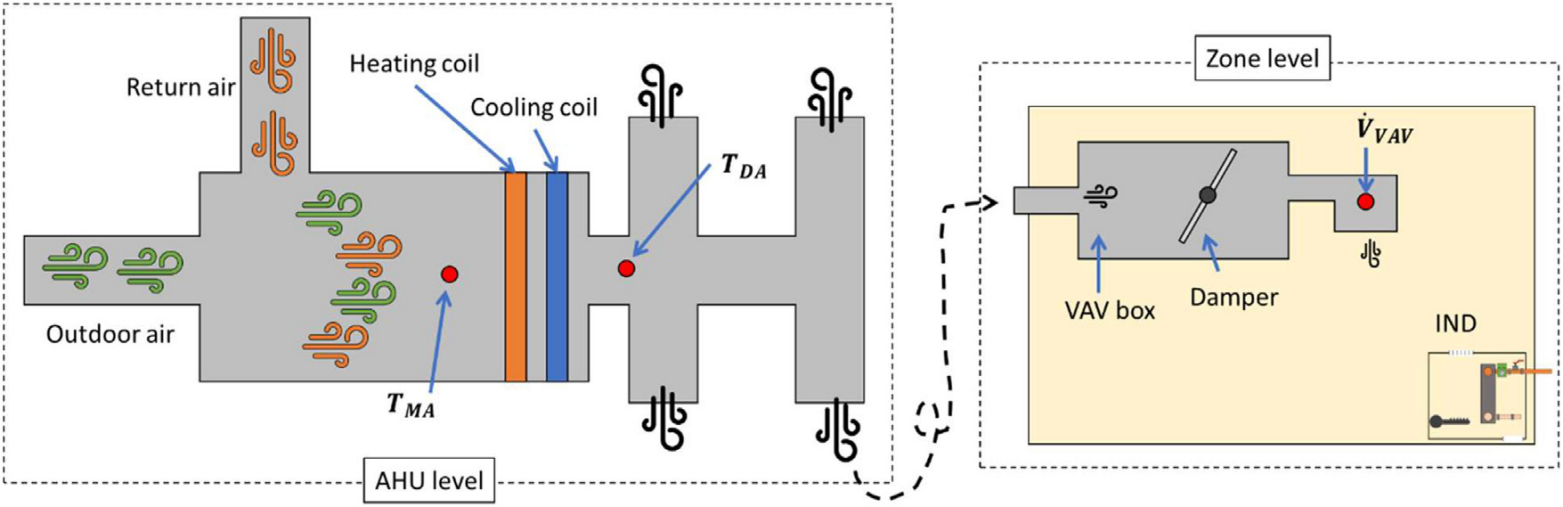
A simplified schematic of the VAV system.

The main AHU connects to VAV systems with no reheat in the study space. For more information, there are four AHUs, and they are connected to each other. The air flow rate of each AHU is 292,230 m^3^/h (172,000 ft^3^/min) with 250 HP and 575 RPM, thus the four AHUs combined provide a total air flow rate of 1168,920 m^3^/h. Since there are four main outlets after processing the air, we measured the differential pressure across the 11 VAV boxes serving the study space using pressure transducers (details are discussed in the Section entitled “VAV instrumentation”) to specify the amount of air coming to the space. We needed to assess the air flow rate through the VAV boxes because only a small portion of the total processed air in the large AHUs is delivered to the study space. In addition, setting a proper ΔT is important because it is one of main drivers to calculate energy consumption and the result can be varied depending on measuring (collecting) points. All the heating and cooling processes in the AHUs occur across the heating and cooling coils. Therefore, we define a mixed air temperature ($T_{MA}$) point located before the coils to represent the mixed outdoor air and return air before it passes over the coils, as shown on the left-hand side of Fig. 8. This point is important, so in a case where the $T_{MA}$ cannot be measured, another approach would be to measure the temperature at the outdoor air stream and return air stream along with the accompanying air flow rates for each stream (or, alternatively, the fraction of damper openings combined with a known mixed or supply air flow rate). After processing the air temperature, a discharge air temperature ($T_{DA}$) is collected as well, which helps quantify the amount of heating or cooling provided. In the AHU for the VAV side, we calculated the HVAC energy consumption using three data points: differential pressure (11 points), $T_{MA}$ (4 points), and $T_{DA}$ (4 points). Some VAVs possibly have a reheat coil inside of the VAV box after the damper, as shown in Fig. 8; in this case, it is important to measure one more point at the outlet of the VAV box, a supply air temperature ($T_{SA}$). We collected $T_{MA}$ and $T_{DA}$ from the BAS since they were available. Fig. 8 shows a schematic of the VAV system and summarizes the data points in the VAV side for the energy calculation. Eq. (3) shows the final form of the VAV energy equation:

(3) $${\dot{Q}}_{VAV}=\dot{V}\times \rho \times C_{p}\times \;\Delta T_{1-2}$$

where $\Delta T_{1-2}$ is equal to $T_{MA}-T_{DA}$ for the cooling mode in summertime while this temperature difference is equal to $T_{DA}-T_{MA}$ for the heating mode in wintertime.

#### Induction units

**Fig. 9:**
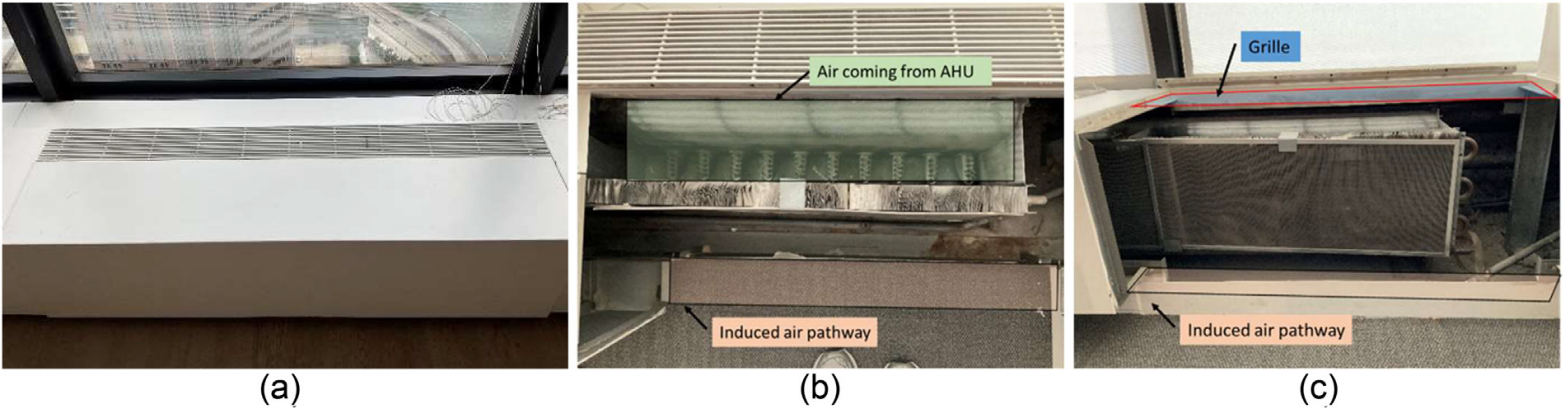
The induction unit system in the test case area: (a) induction unit with the cover, (b) upper view, and (c) the front view.

Induction units, which is a legacy system in the building compared to the VAV system, serve spaces on the North and West, and South and East, with supplying air from seperate AHUs. The configurations of systems are 127,426 m^3^/h (75,000 ft^3^/min) with 147.1 kW (200 HP) and 860 RPM and 141,018 m^3^/h (83,000 ft^3^/min) with 147.1 kW (200 HP) and 860 RPM for these two AHUs, respectively. The measured space faces both South and West, as shown in Fig. 1, and there are two induction units. As shown in Fig. 9(b), which shows induction unit with its cover removed, the induction unit system constitutes from two main parts: (i) the main air discharging part and (ii) the induced air part. Thus, the system provides the processed air from the main nozzle (Green box in Fig. 9(b)) and it induces the air from the room (space) to flow into the induction units.

**Fig. 10:**
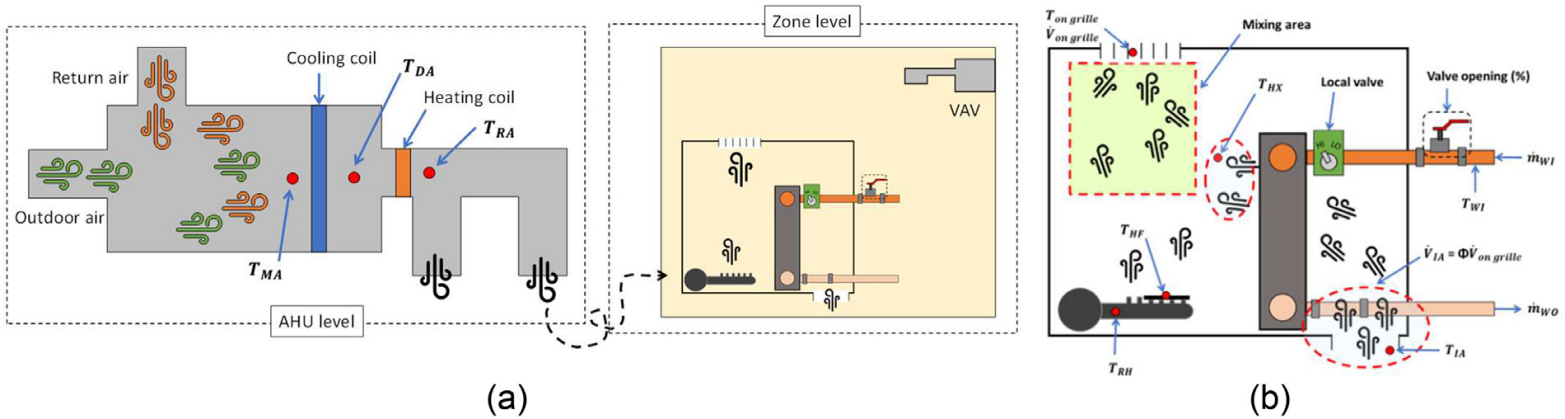
A simplified schematic of the induction unit: (a) inside the AHU and (b) inside the heat exchanger at the zone level.

To measure and quantify the energy consumption on the AHU side of the Induction units, we took a similar approach as we did for the VAV side but added one more point between the coils. Then, we used Eq. (1) to obtain $\dot{Q}$ for after the cooling coil ($T_{MA}$ and $T_{DA}$) and after the heating coil ($T_{DA}\;and\;T_{RA}$), as shown in Fig. 10(a). Also, we needed to characterize one more heat exchange process: the induction unit system, which requires additional information to calculate the energy consumption as shown in Fig. 10(b). An important technical challenge, however, arose from the fact that this system is a legacy system and is only minimally connected to the BAS. More specifically, the BAS provides the percentage of valve opening and the inlet water temperature ($T_{water\;inlet}$), which are both key components to calculate the energy consumption associated with the heat exchanger. However, the water flow rate of coming in and out of the induction unit heat exchanger (${\dot{m}}_{WI}\;and\;{\dot{m}}_{WO}$) and the outlet water temperature ($T_{water\;inlet}$) are required to calculate the energy transfer rate. Additionally, these alone are not sufficient to calculate the energy delivered by the induction unit because occupants also adjust local valves at the floor level, and those are not connected to the BAS and conjecturing the water-out temperature could lead to inaccurate energy estimations. Thus, this study designed an on-site measurement approach to measure the total air flow rate out of the grille at the point after two temperatures, driving air flow and induced air flow, are merged. It is reasonable to assume that there is a mixing area because the induced air passes through the heat exchanger and then mixes with the air from the AHU, as shown in Fig. 9(b) and (c) and Fig. 10(b). In other words, the direction of the induced air is from the bottom of the induction unit and passes through the heat exchanger and then gets mixed with the air from the AHU that is supplied to the space through the nozzles. The best practice here is to measure the water flow rate, ${\dot{m}}_{WI}\;and\;{\dot{m}}_{WO}$, and measure the temperature of these two points. However, it would have required a costly and intrusive measuring process in this project, which was not appropriate for the test case and study space.

The air temperature on the grille ($T_{on\;grille}$) is the terminal air temperature delivered to the space. Assuming the air from the nozzles and the induced air are independent of each other, the temperature after the heat exchanger ($T_{HX}$) can be calculated as shown in Eq. (4). We measured the air velocity at the induced air pathway and on the grille with a handheld anemometer, Extech AN100 [51]. By multiplying the area of each opening, the induced air flow rate (${\dot{V}}_{IA}$) and the air flow rate on the grille (${\dot{V}}_{on\;grille}$) are estimated and used to establish the fraction of the total air flow rate that is attributable to the induced air flow rate (ϕ). The values are 37 % and 27 % of the total air flow rate for the South and West sides, respectively. Then, known parameters including the temperature on the grille ($T_{on\;grille}$), the temperature on the nozzle ($T_{HF}$), the air flow rate from the induction units (${\dot{V}}_{on\;grille}$), and the fraction of induced air (ϕ), are used to calculate the temperature at the in-unit heat exchanger ($T_{HX}$). This equation is exclusive to this experimental design due to the limited access to the variables (i.e., ${\dot{m}}_{WI}$, ${\dot{m}}_{WO}$, $T_{WI}$, and $T_{WO}$). It is highlighted that this approach is subject to be modified in a future study in other buildings, depending on the specifics of a different building.

(4) $$T_{HX}=\left(T_{on\;Grille}-T_{HF}+\phi_{Side}T_{HF}\right)/\phi_{Side}$$

Energy calculations of the induction units for the AHU side and Zone level (heat exchanger), were conducted by using the fraction of induced air (ϕ). The final form of the induction units’ energy equations is presented in Eq. (5) and (6)

(5) $${\dot{Q}}_{IND,AHU}=\left(1-\phi \right)\times {\dot{V}}_{on\;grille}\times \rho \times C_{p}\times \;\Delta T_{1-2}$$

where, $\Delta T_{1-2}$ is $T_{MA}-T_{DA}$ for the cooling mode (summer) and $T_{RA}-T_{DA}$ for the heating mode (winter).

(6) $${\dot{Q}}_{IND,Zone,HX}=\phi {\dot{V}}_{on\;grille}\times \rho \times C_{p}\times \;\left(T_{Zone}-T_{HX}\right)$$

where, a zone air temperature, referring as induced air temperature, is $T_{Zone}$, and $\Delta T_{1-2}$ is $T_{Zone}-T_{HX}$ for the cooling mode (summer) and $T_{HX}-T_{Zone}$ for the heating mode (winter).

#### Fan system

A reverse sizing exercise was conducted to estimate fan energy consumption by the AHUs because there was limited information on fan performance in the AHUs on site. For the sizing exercise, we set a size of fans and used eCAPS to find the proper size of fans based on the maximum CFM and the static pressure. eCAPS is the online selection toolbox for fan, louver, make-up air, energy recovery, dedicated outdoor air systems (DOAS), damper, grille, register, diffuser, and air terminal unit developed by Greenheck [52]. We then used fan affinity laws, shown in Eq. (7), to calculate the fan energy consumption [53]. It is highlighted that in this process we discovered several discussions and papers about the nature and magnitude of the exponent in the power-law relationship between power and flow in real-world applications [[54], [55], [56], [57], [58]]. These studies include:

(1) A study conducted by the Pacific Northwest National Laboratory (PNNL) suggests using exponential as 2.5 for the power-law fan model [54].
(2) Conversations between engineers questioning the utility and reliability of using exponent 3.0 in the real-world application [55].
(3) A study that highlighted that many engineers are trying to use the exponent between 2.x, where 0 < *x* < 9 to overcome a theoretical (ideal) assumption [56].
(4) An article in the ASHRAE Journal mentioned that in the case of not operating with a full load (50 %), the author does not prefer to use 3.0 because he believes it is more likely to be closer to 2.0 (square of flow) [57].
(5) A recent journal article in ASHRAE Journal highlighted 2.7 as an appropriate exponent when Variable Frequency Drive (VFD) speed and input power ranged from 40 to 100 % [58].

In reviewing these existing studies and further discussions with an energy industry professional, the present study determined to calculate the fan energy consumption with 2.5, which is a conservative approach, as shown in Eq. (8).

(7) $$W_{1}=W_{2}{\left(\frac{Q_{2}}{Q_{1}}\right)}^{3}$$

(8) $$W_{1}=W_{2}{\left(\frac{Q_{2}}{Q_{1}}\right)}^{2.5}$$

where, W is a brake horsepower (kW), and Q volume airflow rate (m^3^/s).

A full brake horsepower (BHP) load (kW) is a power output of an engine by a measurement and it means an actual usable power coming from the engine, which is more accurate than horsepower (HP) [59]. In this study, the BHP was estimated considering a fan efficiency of 60 % and a motor efficiency of 90 %. The fan energy consumption calculation required the assumption due to a discrepancy between the desired data (i.e., air flow rate in the test spaces) and the available data (i.e., total air flow rate in AHUs). However, the best practice in this calculation is to use the fan specifications when the fan system is specified to (or properly split into) the target spaces.

## Design and selection of instrumentation for data collection

This section summarizes the selection and installation of the instruments and procedures for data collection from the existing BAS needed to utilize in the equations mentioned in the previous section.

### VAV instrumentation

**Fig. 11:**
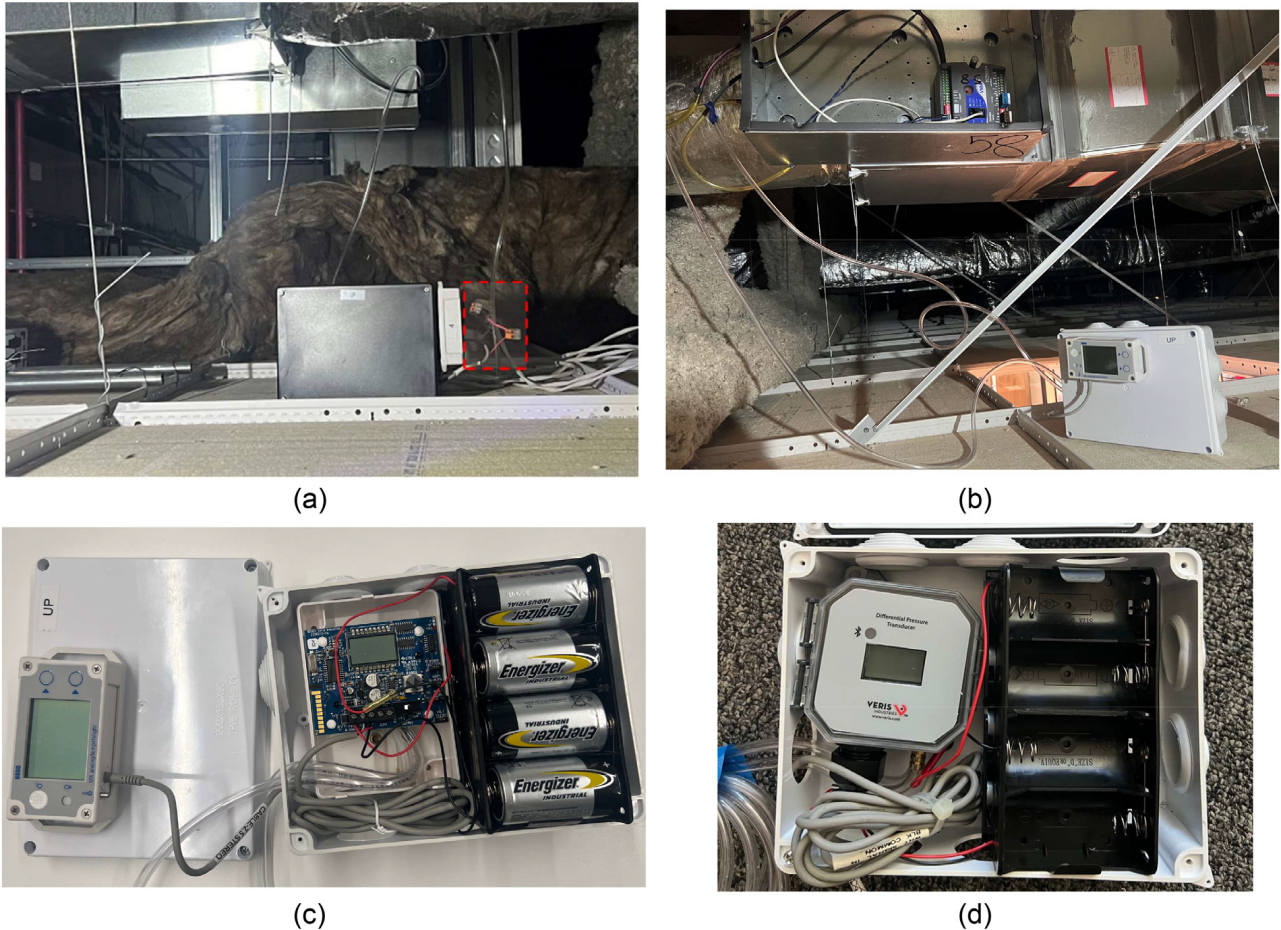
The customized differential pressure measuring box in the drop ceiling: (a) wired by a low voltage power line and (b) battery powered custom boxes, (c) T-VER-PXU-L, and (d) T-VER-PX3UL connected to MX1105 logger as a 0–5 VDC.

Calculating heating and cooling associated with the VAV system requires relying on air flow rate, temperature, and relative humidity (RH) of each VAV box delivered to each thermal zone. However, relative humidity was not used in the calculation since the scope of the calculation is the sensible heat transfer. There are eleven VAV boxes in the space. The initial idea of this set-up is that all sensors and loggers are connected with a low voltage power supply distribution, which guarantees a constant power supply to all sensors and loggers, and meets the need for minimal intrusion to the space. However, this approach was not possible when the installation began. To measure air flow rate in each VAV box, we built 11 custom-made boxes (similar to a previous study [60]) to accommodate Veris differential pressure transducers (T-VER-PXU-L [61] and T-VER-PX3UL [62]) connected to Bluetooth enabled MX 1104 [63] and/or 1105 data loggers [64] with a DC voltage input cable [65]. Both MX 1104 and MX 1105 are suitable options since one external channel is needed. MX 1104 measures temperature, relative humidity, and light level and has only one external channel. The MX 1105 loggers do not measure any variables, but they have four external channels. To connect the pressure transducer to the pressure taps before and after the VAV boxes, a short but sufficient length of PVC – ¼ inch vinyl plastic tubing was used. Most of the VAV boxes have a vacuum connector, so the readings could be both used for the study design and the BAS sensor and the installed pressure transducer. When the connector was not already installed, a new vacuum connector was installed. Due to challenges in maintaining a stable voltage reading, we were able to use the low voltage connection only for two of the 11 customized boxes shown in Fig. 11(a). The small red dotted box shows the low voltage connection to supply power. The other 9 custom boxes were powered by a pack of 8 D batteries (i.e., a total of 12 VDC), shown in Fig. 11(b).

In addition, we considered two different versions of Veris pressure transducers, including older T-VER-PXU-L and newer T-VER-PX3UL, based on availability and tested for differences in their performance. Based on the manufacturer’s datasheet, the differential pressure transducers have a linear response to voltage output [61,62]. Fig. 11(c) and (d) show inside of the custom boxes operated by 8 D batteries. For this study, given the range of differential pressure across the VAV box, the range is set to 0 ‒ 5 VDC which is equivalent to 0 to 622 Pa (0 to 2.5 in w.c.). For the transducer, T-VER-PXU-L, we adjusted the dial (shown the black triangle on the knob in Fig. 11(c)) on the circuit board shown in Fig. 11 to the desired pressure range. The pressure transducer has several ranges that set the maximum measurement limit to 25, 62, 125, 250, 622, or 2,488 Pa (0.1, 0.25, 0.5, 1, 2.5, 5, or 10 in w.c.). It is important to ensure that the range was set correctly, otherwise high values would exceed maximum limits and not yield useful data or low values would be read at too low resolution to be useful.

Several approaches could be taken to ensure the correct range is selected: (i) review the BAS trend data if available, (2) use a reference pressure transducer that is not dependent to the pressure range, (3) conduct a flow hood measurement of the diffusers connected to the VAV box, or (4) review the VAV testing and balancing report if available. Some iteration may be needed. The data from 11 transducers and 22 data loggers were captured in real-time for this study. Furthermore, it is noted that for the quality assurance and quality control (QA/QC), we followed a three-step process to confirm our sensors’ life span and reading quality before deploying this box or after replacing batteries.

As a final process to calculate the air flow rate (m^3^/h or ft^3^/min), the measured differential pressures are converted to an air flow rate using Eq. (9) [66]. It this equation, CFM represents the air flow rate and is converted to unit of m^3^/h, K is a conversion factor depending on the size of the ducts and manufacturer, specifically, it is a flow having unit of CFM, which produce 248.84 Pa (same as 1.0 in w.c.), and ΔP is a measured differential pressure having unit of in w.c.

(9) $$CFM\;\left(\frac{ft^{3}}{min}\right)=K\sqrt{\Delta P}$$

At the zone level, the 11 diffusers connected to the 11 VAV boxes were monitored to measure supply air temperature and relative humidity using Onset MX 1104 data loggers [63]. Figure S1 shows the sensor installed in parallel to the U-12 HOBO loggers [67]. Early on, for a few ceiling diffusers and for a few weeks, the loggers were installed in parallel for QA/QC purposes. The loggers were hidden from the occupants as they were installed immediately after the air supply between above the face cover.

Table 1 summarizes the instruments used in the measurement of the VAV system. The table includes the make and model of each sensor, the parameter that was the primary variable to measure, the unit of the measurement, the number points in the system/space in which the instruments were placed, the location of the instrument installation, the frequency (time interval) of the data recording, and the resolution and accuracy of the instruments. N/A for the equation means that they were used for the QA/QC primarily and the recorded values were not used in the final calculations. However, except for light intensity, other measurements were used in the QA/QC process even though they are not directly used in any of the equations.

**Table 1:** A detailed summary of the instruments used for the VAV system measurements: make and model, measured parameter, number of data acquisition points, installed location, frequency (time interval) of measurements, applied equation, and resolution as well as accuracy of the recorded data.

Model	Parameter	Unit	# of points	Location	Time interval (min)	Equation	Resolution (Accuracy)
Veris T-VER-PX series	Differential pressure	Pa	11	VAV box	5	(1) and (7)	N/A (1 %)
Onset MX1104 & MX1105	Temperature	°C	11	VAV box	1	N/A	0.002 °C at 25 °C (±0.20 °C in range of 0 to 50 °C)
	Relative humidity	%	11	VAV box	1	N/A	0.01 % (±2.5 % in range of 10 to 90 %)
Onset MX1104	Light intensity	lux	11	Diffuser	1	N/A	N/A (10 %)
Onset U12	Temperature	°C	11	Diffuser	1	N/A	0.03 °C at 25 °C (±0.35 °C in range of 0 to 50 °C)
	Relative humidity	%	11	Diffuser	1	N/A	0.05 % (±2.5 % in range of 10 to 90 %)
	Light intensity	lux	11	Diffuser	1	N/A	N/A (N/A)

### Induction unit instrumentation

**Fig. 12:**
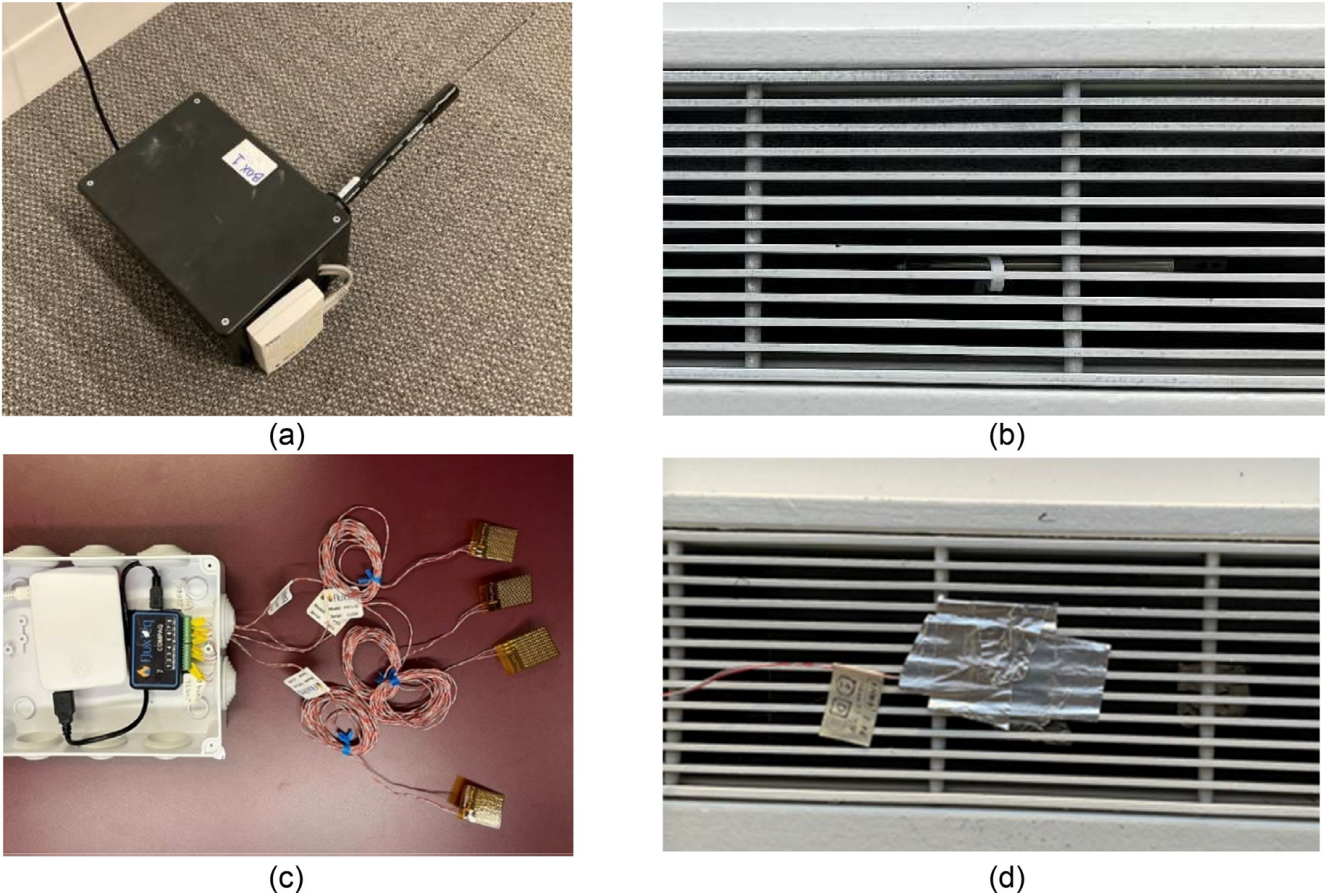
(a) The custom enclosure for utilizing air velocity and temperature sensors, (b) installation of the velocity sensor in the discharge grille of an induction unit, (c) the completed set of heat flux sensor box and (d) installation of the heat flux sensor on the grille.

Similar to the VAV system, to calculate heating and cooling energy consumption of the induction unit system that serves the perimeter zones, a set of monitoring instruments was deployed. Specifically, due to the complex nature of the induction unit system that requires monitoring both the hydronic system and the air system, this study developed a solution that utilized enough instruments to monitor the conditioned air at the discharge grille rather than monitoring the hydronic and the air system separately as explained in Eq. (4). Consequently, this study measured the air velocity and air temperature at the discharge grille, as shown in Fig. 12. This figure illustrates the installation of the air velocity sensors, air temperature sensor, and heat flux sensors. One T-DCI-F350-W5 × 3 [68] and two T-DCI-F300–1B3 [69] air velocity sensors were installed in the induction units, and the calculated area of the grille opening (measured once) is multiplied by the air velocity to yield air flow rate. The T-DCI-F350-W5 × 3 required only one external channel while the T-DCI-F300–1B3 required two external channels to measure temperature in addition to velocity. For both sensors, an additional surface temperature sensor was used to also measure the air temperature from the grille [70]. These four sensors provided a good understanding of the air flow patterns in the induction units.

We used a combination of a heat flux sensor (FluxTeq PHFS-01 [71], shown in a thin golden film in Fig. 12(c)) and a data acquisition system (FluxTeq COMPAQ [72], shown in a small black box in Fig. 12(c)). This sensor configuration was designed to measure the heating and cooling output from the induction units installed on the perimeter zones. Fig. 12(d) shows each use case, respectively. We collected the data, which is the supply air temperature coming from the induction units at the grille level. It is noted that the accuracy of heat flux and temperature data from the FluxTeq depends on the thermal (contact) resistance. In this study, we used the reported uncertainty (accuracy) by assuming the data follows the same uncertainty. The data acquisition system was connected to a Raspberry Pi with custom-made Python scripts collecting temperature every minute. We deployed five sensors; three are for the induction units and the remaining two are for the shading device. The heat flux sensors were attached to the induction unit and the windows using 3 M High Temperature Flue Tape [73].

Similar to Table 1, Table 2 summarizes detailed information for the induction unit system instrumentation. The heat flux instruments were also used for the shades to measure the heat flux at the surface of the windows and shades in one south-facing and one west-facing shade. A similar approach to measure additional variables for QA/QC purposes were conducted for the induction unit system too. Some of these measurements were spot measurements and not continuous measurements.

**Table 2:** A detailed summary of the instruments used for the induction unit system and shades: make and model, measured parameter, number of data acquisition points, installed location, data collection frequency (time interval), applied equation, and resolution as well as accuracy of the recorded data.

Name	Parameter	Unit	# of points	Location	Time interval (min)	Equation	Resolution (Accuracy)
T-DCI-F3 series	Air velocity	m/s	3	Grille	1	(1), (4), (5), (6)	N/A (± 4 % in range of 0.5 to 10 m/s)
TMCx-HD w/ MX1104	Temperature	°C	3	Grille	1	(1), (4)	0.002 °C at 25 °C (± 0.15 °C in range of 0 to 70 °C)
Extech AN100	Air velocity	m/s	2	Induced air part	Instantaneous	(4)	0.01 m/s (± 3 %)
DIGI-SENSE 20,250–22	Air velocity	m/s	6	Grille	Either 10, 1, or 5	N/A	0.01 m/s (± 3 % + 0.2 m/s)
U12	Temperature	°C	6	Grille	Either 10, 1, or 5	N/A	0.03 °C at 25 °C (±0.35 °C in range of 0 to 50 °C)
	Relative humidity	%	6	Grille	Either 10, 1, or 5	N/A	0.05 % (±2.5 % in range of 10 to 90 %)
	Light intensity	lux	6	Grille	Either 10, 1, or 5	N/A	N/A (N/A)
FluxTeq PHFS-01	Heat flux	W/m^2^	10	Nozzle	1	N/A	up to 2 W/m^2^ (up to ± 5 %)
			3	Shade	1	N/A	
			4	Window	1	(9)	
	Temperature	°C	10	Nozzle	1	(4)	N/A (± 1.0 °C discussed in “Propagation of Uncertainty in Energy Consumption Calculations”)
			3	Shade	1	(9)	
			4	Window	1	(9)	

### Design and integration of the instruments and the data collection process

Prior to the data collection, we tested the system, instruments, and the shades in operation for a few months to resolve as many issues as possible before the study officially started. The selection criteria for the sensors and loggers were: (1) remote transmission of data transmissions and upload of data, as much as possible, (2) built-in data storage in addition to the remote transmission to avoid loss of data, (3) accuracy and reliability of the sensors to operate with low maintenance, (4) a nonintrusive approach to the case study space both during and after the study project is completed (i.e., minimal penetrations or other aesthetic changes), (5) long-term data storage capability to ensure the loggers and sensors, (6) ease of data download, (7) functionality for a multi measurement capability of each sensor, and (8) if possible, allowing redundant data collection for QA/QC. Overall, the best practice for the sensor selection is to meticulously select and, in some cases, build custom solutions, and configure the remote data transmission with online platforms, if possible. Adding redundancy is always recommended to ensure there is no data loss. The details of QA/QC process during the measurement are discussed in the Supplementary Material.

**Fig. 13:**
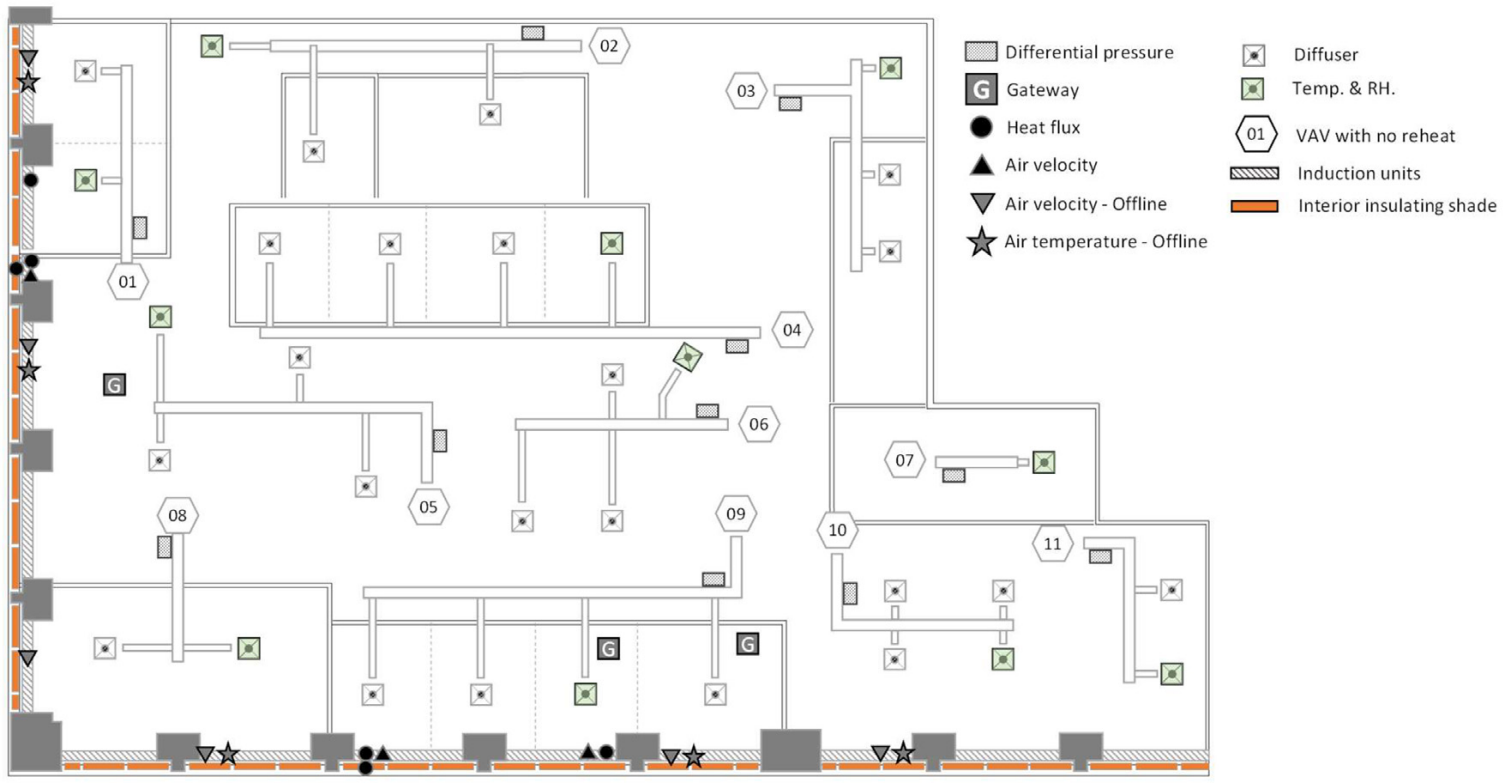
The locations of sensors and set-ups in the test space for the Onset HOBO data loggers, offline sensors, and customized heat flux sensors, excluding the sensors related to the Amatis system.

One of the highlighted features in this configuration is that the sensors are automated to transmit real-time data to a cloud-based storage system. Three different platforms were utilized. The first is the Onset HOBO cloud in which the air temperature, relative humidity, air velocity, and differential air pressure sensors were connected to MX Gateway (MXGTW1) [74]. Fig. 13 shows the data acquisition points for the Onset HOBO Data Loggers, the heat flux, and offline sensors (i.e., Anemometer: DIGI-SENSE and temperature: HOBO U12). There are 11 differential air transducers shown in dotted boxes in Fig. 13, and 11 measuring spots to retrieve the data of temperature and relative humidity at the diffuser level shown in Fig. 13. The customized differential air measuring boxes connected to the gateway via MX 1104 (or 1105). There is one more HOBO configuration we used in the induction units for measuring air velocity and air temperature, shown in black triangles on the induction units in Fig. 13. This configuration also used connection with MX 1104 (or 1105) to transmit real-time data to the cloud. The gateway’s locations are expressed in the box with capital letter “G” in Fig. 13. Since MX 1104 and 1105 are able to transmit data up to 30 m, the distribution of gateways captured all data from the test space. In this platform the data is transmitted to the cloud system provided by Onset [75]. For redundancy, there are 12 auxiliary sensors for air velocity and air temperature at the induction units expressed in upside-down gray triangles and gray stars in Fig. 13, respectively. These were not connected to a cloud system and required manual download.

The second platform is for the heat flux sensor configuration; it is different from the Onset platform and was developed for a prior project [76]. Black circles in Fig. 13 show the locations where we deployed the heat flux sensors to measure the heat flux (W/m^2^) through the glazing and shades and the induction units. This configuration used a collection of Raspberry Pis to broadcast real-time data to Grafana [77], a real-time visualization dashboard [78,79]. The dashboard was built to collect temperature and heat flux from the heat flux sensors and allows bulk downloading and reporting statistics for each channel. Originally, the data was transmitted to a server on the Illinois Tech campus and over time the short-term data was stored for trending on the Digital Ocean platform on the back end and on the internal server for long-term data storage.

**Fig. 14:**
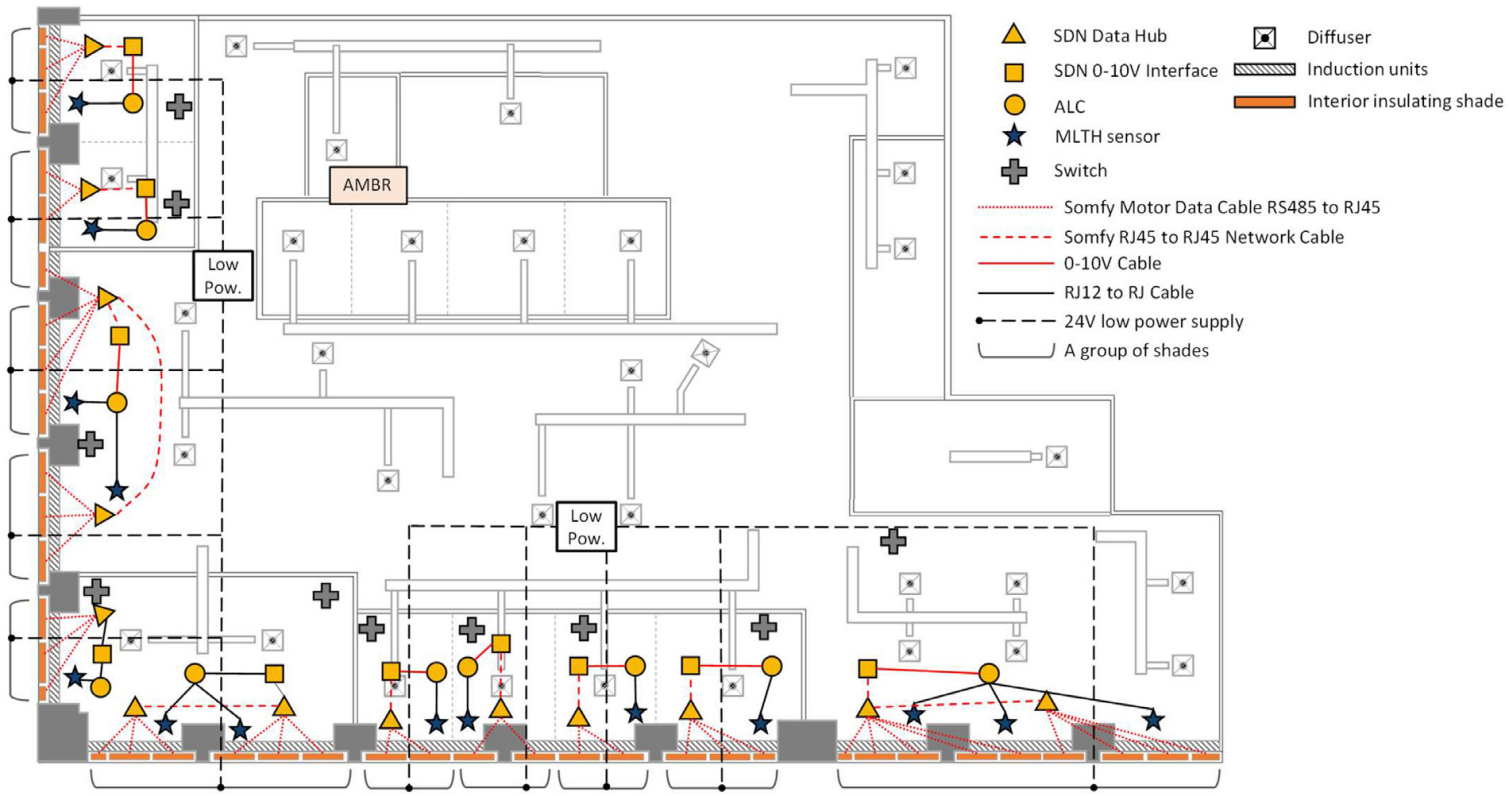
The location of the sensors and set-ups in the study design space for the Amatis platform.

Fig. 14 shows the locations of instruments and set-ups with the third platform, Amatis. The gray bracket shows each control group based on control sequences or preference. The SDN Data Hub connected to the actuator to adjust the position of shades and the signal coming from a higher level, the SDN 0–10 V Interface followed by the ALC. MLTH sensors connected to the ALC, which communicates with the AMBR. To provide the low-voltage to the actuators, we installed two low-voltage power supplies as shown in Fig. 6, and each supply distributes the power to designated shades groups. Finally, Amatis Border Router transmits the real-time data to the Amatis Energy Dashboard [37].

The last platform is the building’s BAS system, from which some data were collected. For most of the points indicated with a certain status indicator, with a mouse click it was possible to see the current data that is being recorded. Some of the trend data such as the VAV flow rates were collected for about a year while other data were mostly recorded up to a week or up to a month. The BAS was set up by the building operator and this study coordinated with the building engineers to extract the data and conduct QA/QC for the accuracy of the sensors or replacements when needed. The challenges with the BAS data collection were: (1) it was a tedious process to click on the GUI and manually download the data one by one as no API for fast and bulk download was available to the study team and (2) the data for some variables were not collected on a long-term basis, requiring frequent intervention to download data.

**Fig. 15:**
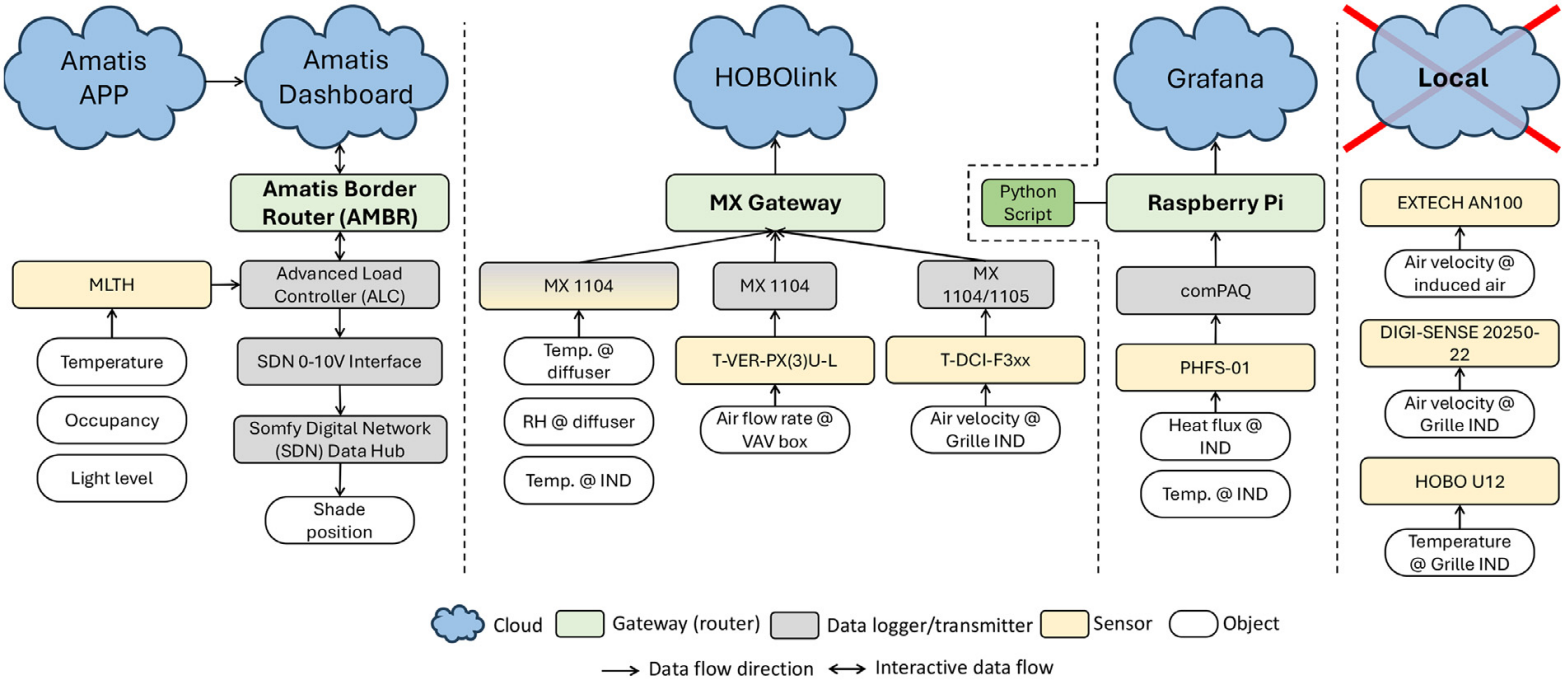
The configuration of data flow, cloud systems, and local systems used in this study.

Fig. 15 summarizes sensor configurations. The color coding and the direction of the arrows in the figure shows additional information about the type of the instruments and the data flow, respectively. We facilitated three cloud systems to obtain the data for this study, excluding the remote BAS access. Some local sensors were also used to obtain additional data for QA/QC and the data was collected every few weeks. The Amatis App allows a control algorithm in the system such as Dynamic control strategy, and this concept can be developed to be a more interactive closed loop system for a dynamic shading device. The system also offered an advanced MQTT (Message Queuing Telemetry Transport) protocol that was tested but not implemented for this study as it was under development for the full functionality. The Amatis system was connected via a hotspot and there was no issue with the wireless connectivity during the study.

The MXGTW1 Gateway allowed utilizing the HOBOlink cloud system to transmit the data and store the data. In addition, the HOBOlink cloud system allowed setting up trends, automated data export, and several advanced features. The gateways were connected through the building Wi-Fi. Several times, every few weeks, one gateway lost connection with the Wi-Fi and the Wi-Fi setting had to be reset. It was unclear if the Wi-Fi protocol did not have the same capability with the gateway protocol. The gateway was able to transmit the data during offline mode even though the gateway was connected to the internet after a few days. In addition, the MX loggers have built-in data storage in addition to the cloud connection which allowed them to collect all the data even during the period that the gateway was offline. One important detail is to optimize the location of the gateways, so all the loggers could send the data without any issues. Often the recommended distance in the gateway data sheet needs to be shortened as there are blockages by objects (e.g., ceiling tiles and furniture) and the building structure (e.g., steel frame vs masonry frame) that could impact the range. Originally two gateways were installed for this study but over time an additional gateway was installed to ensure the loggers at the edge of connectivity are connected all the time.

The third cloud system was built by the study team using the Grafana interface. Python scripts were developed to run on the Raspberry Pi and to transmit the data to the cloud, available here: [80]. The heat flux instruments were connected to the low voltage line as previously described. A few times the connection was interrupted by several factors: (1) losing connection to the Wi-Fi and (2) changing the transmission port. The script was updated over time to be more robust and address these interruptions. During the transmission issue and after the Wi-Fi connection was established the heat flux sensors did not transmit the offline data. After the study was over the secure digital (SD) cards connected to the Pis entailed all the data and those missing data were retrieved.

Lastly, we used the existing BAS to download data and to calculate energy consumption associated with the HVAC system. The building has a built-in BAS, Metasys provided by Johnson Controls, and provides some of the essential data to calculate HVAC energy consumption not only AHU level but also zone level. We collaborated with the building engineers to understand the current BAS and download the necessary data from the BAS to be either used for the calculations or as an extra QA/QC process. Detailed information about the Amatis and the BAS systems is summarized in Table 3.

**Table 3:** A detailed summary of the instruments used for the Amatis and BAS system: make and model, measured parameter, number of data acquisition points, installed location, data collection frequency (time interval), applied equation, and resolution as well as accuracy of the recorded data.

Name	Parameter	Unit	# of points	Level	Time interval (min)	Equation	Resolution (Accuracy)
MLTH (Amatis)	Temperature	m/s	14	Zone	1	N/A	N/A (± 0.5 °C in range of 15 to 40 °C)
	Relative humidity	lux	14	Zone	1	N/A	N/A (± 3.5 % in range of 20 to 80 %)
	Lighting level	%	14	Zone	Instantaneous	N/A	N/A (N/A)
	Motion (room occupation)	%	14	Zone	Either 10, 1, or 5	N/A	N/A (N/A)

BAS (Metasys)	Reheat air temperature	°C	2	AHU for IND	10	(1)	N/A (± 0.5 °C discussed in “Propagation of Uncertainty in Energy Consumption Calculations”)
	Mixed air temperature	°C	2	AHU for IND	10	(1)	
	Discharge air temperature	°C	2	AHU for IND	10	(1)	
	Mixed air temperature	°C	4	AHU for VAV	10	(1)	
	Discharge air temperature	°C	4	AHU for VAV	10	(1)	
	Zone air temperature	°C	11	AHU for VAV	30	(1) and (6)	
	Mode	Summer / Winter	2	AHU for IND	30	(3), (5), (6), and (9)	N/A (N/A)

## Propagation of uncertainty

Propagation of uncertainty for a function is a quantitative assessment on how uncertainties corresponding to various variables and parameters (i.e. measurement error, etc.) propagate and affect the final result (i.e., HVAC energy consumption and savings under different control strategies). Propagation of uncertainty should be addressed to consolidate the findings by showing the impact of the assumptions and measurements total error. Propagated uncertainty for measured parameters depends on both the accuracy of the sensors and the proficiency of the performer; the former is quantifiable while the latter is less so. To minimize the latter effects, we ensured that there is a sequence of procedures (SOP) in place to minimize the uncertainty associated with the performer conducting the measurements. This allowed us to use the same procedures with the same sensors in the same measuring locations to the greatest extent possible [81].

To propagate uncertainty for a function that uses measurement values from multiple sensors, uncertainty corresponding to each sensor must be known and used. A total of 44 data points were collected from various sensors and used to obtain energy consumption. These data points include: (1) BAS data, including 14 air handling unit temperature values before and after the central heating (or cooling) coils, two operational modes (i.e., heating and cooling), 11 zone air temperatures for the induction units, and (2) measurement data, including 11 differential air pressure values for VAV boxes, three air velocity values for the induction units, and three air temperature values for the induction units. By using many sensors, the uncertainty from each instrument propagates through the calculation process and impacts the final results [82]. As it was described in the study design, there are two main systems: the VAV and induction units (see Fig. 1), and the systems provide a processed air from each AHU system. Furthermore, the induction units have the zone level heat exchanger inside of the units.

In this study, we considered two different methods to propagate uncertainty for HVAC energy consumption, including (1) conventional and (2) Monte Carlo. The uncertainty quantifications result from both the equations used to obtain energy consumption and the utilized sensor’s reported accuracy, error, or standard deviation, as applicable. The main reason we implemented two methods is to explore the potential differences caused by each method, and review how uncertainty propagates in the system.

### Propagation of uncertainty: conventional method

In order to calculate propagated uncertainties in HVAC energy consumption, we used the conventional method by applying two rules of quadratic sums of absolute and relative errors based on the underlying equations [82]. This calculation method assumes that reported sensor uncertainties follow normal distributions. Propagation of uncertainties was calculated in each equation using accuracy of sensors, and quadratic sums of absolute and relative errors, respectively [83,84], following Eqs. (10) and (11).

(10) $$U_{z}=\sqrt{{U_{x}}^{2}+{U_{y}}^{2}+\cdots }$$

(11) $$\frac{U_{z}}{z}=\sqrt{{\left(\frac{U_{x}}{x}\right)}^{2}+{\left(\frac{U_{y}}{y}\right)}^{2}+\cdots }$$

where $U_{z}$ is the absolute uncertainty of parameter z, taken as the quadratic sum of $U_{x,\;y,\;and\;more}$, which is the accuracy of each sensor measurement, $\frac{U_{z}}{z}$ is the fractional uncertainty in the measurement, taken as a quadratic sum of each sensors’ relative or fractional uncertainties, and x(ory) is the measured value.

Eq. (1) is used as the main equation to calculate energy consumption in the AHU for heating and cooling end-uses in the space. Eq. (12) describes the simple form of equation to calculate the propagation of uncertainty in the energy consumption calculation, assuming the density and the specific heat values are constant [84]. This equation can be varied and applied depending on demand.

(12) $$\frac{U_{\dot{Q}}}{\dot{Q}}\left(\%\right)=\sqrt{{\left(\frac{U_{\dot{V}}}{\dot{V}}\right)}^{2}+{\left(\frac{U_{T_{before\_coil}}}{T_{before\_coil}}\right)}^{2}+{\left(\frac{U_{T_{after\_coil}}}{T_{after\_coil}}\right)}^{2}}$$

where, $U_{\dot{Q}}$, $U_{\dot{V}}$, $U_{T_{before\_coil}}$ and $U_{T_{after\_coil}}$ are the absolute uncertainty of $\dot{Q}$, $\dot{V}$, $T_{before\_coil}$, and $T_{after\_coil}$.

It is important to note that the final uncertainty we sought is based on a fractional (or relative) format in (%), not absolute (e.g., ± 1.5 °C), and thus, the temperature values are important to convert to the fractional form shown in Eq. (11). Other values we used provide errors in the fractional format. The main reason we focused on fractional uncertainty is to reflect the proportional significance of uncertainty in the system and to be able to estimate propagated uncertainty resulting from different types of measurements.

For the heat exchanger energy consumption calculation, it is more difficult for the heat exchanger, inside of the induction units, because there is limited access to the system referring to Eq. (4). The process we applied for $U_{T_{HX}}$ was to break down each component as shown in Eq. (4). To calculate $U_{T_{HX}}$, we calculated the uncertainty for the first component $\phi_{Side}$, expressed in $U_{\phi_{Side}}$. The next step was to calculate the uncertainty for the component of $\phi_{Side}T_{HF}$, expressed in $U_{\phi_{Side}T_{HF}}$, which is the propagated uncertainty from $U_{\phi_{Side}}$. The third step was to calculate uncertainty for components inside of parentheses in Eq. (4), expressed in $U_{T_{on\;Grille}-T_{HF}+\phi_{Side}T_{HF}}$. Finally, we calculated $U_{T_{HX}}$ according to Eq. (4).

Since the access to the main AHUs was limited and thus sensors could not be deployed within, and because sensors were already installed by the manufacturer inside the AHUs and stream data to the BAS, the AHU energy analysis relies on the temperature values from the BAS. The ideal approach to understand measurement uncertainty would be to review the manufacturer data sheets for the temperature sensors used inside of the AHUs. However, this information was missing in our study, so we reviewed the likely range of measurement uncertainty of typical temperature sensors. There are mainly three types of temperature sensor (i.e., thermistors, resistance temperature detectors (RTDs), and thermocouples) [[85], [86], [87], [88]]: (1) thermistors work based on a thermally sensitive resistor. The output temperature value is calculated using a temperature-resistance curve. The uncertainty of a typical thermistor is around ± 0.2 °C and the high quality one is around ± 0.1 °C. (2) RTDs are a type of sensor that is a metal-based temperature sensor. Platinum is known for the metal used for this sensor (Pt1000). It is the most stable and accurate among other types; however, it is expensive and fragile. The typical uncertainty of this type is around ± 0.1 to ± 0.3 °C, depending on the temperature range. (3) thermocouples use two metal probes at the measuring end in a junction and calculate the temperature based on a generated voltage between two probes based on a correlation. It is relatively inexpensive and less accurate compared to other types. Type K is widely used, and its uncertainty is around ± 2.2 °C or ± 0.75 %, whichever is greater, and in case of Type T, it follows that ± 1.0 °C or ± 0.75 %, whichever is greater.

A prior study collected the uncertainties of such measurements, as summarized in Table 4 [81]. Other studies designated the uncertainty of the temperature sensor in HVAC systems as ± 0.5 °C [89] or ± 0.1 °C [90]. For the accuracy of temperature data in the BAS system and temperature at the outlet of the induction units, we assumed it as ± 0.5 °C, $U_{BAS}$. Based on the report of the heat flux sensor, it has Type T, and we regarded the uncertainty as ± 1.0 °C, $U_{T_{HF}}$. It is important to note that in this analysis, it ignores the uncertainty introduced by the unit conversion (e.g.,°F to °C) and assumes that the constant variables do not create additional uncertainty.

**Table 4:** Summary of uncertainties of measured temperature values in [81].

(± °C)	Lab setting	Measurement & Verification (M&V)	Verified Service Providers (VSP)	Contractor Current	Architectural Energy Corporation (AEC) 2004	Architectural Energy Corporation (AEC) 2010
Supply Air	0.65	0.19	0.61	1.17	0.83	0.83
Return Air	0.51	0.27	0.61	1.17	0.83	0.83
Outdoor Air	0.51	0.33	0.61	1.17	0.83	0.83

### Propagation of uncertainty: Monte Carlo method

For the second method to uncertainty propagation, we implemented Monte Carlo simulation, which uses a statistical method to estimate uncertainty of the result by assuming that all possible measurements of a variable form a normal distribution [91]. In every iterative sequence, we calculated the output’s mean and standard deviation, $\bar{\dot{Q}}\;$and $\sigma_{\dot{Q}}$. By having both output mean and standard deviation, one can simply estimate uncertainty. This calculation regards all parameters as independent. The calculation process of Monte Carlo uncertainty analysis is as follows:

1) Consider a normal distribution for each measured parameter where its mean is equal to actual measured value and its standard deviation is equal to measurement accuracy (e.g., $U_{i}$).
2) Randomly select a value from each normal distribution corresponding to the measured parameter.
3) Repeat Step 2) up to a designated number of iteration to generate the random samples (here, 1,000 iteration is considered) [92].
4) Use randomly selected values from Step 3) to calculate ${\dot{Q}}_{i}$, as such, the VAV has two parameters ${\dot{V}}_{i}$ and $\Delta {T_{1-2}}_{i}$ according to Eq. (3). There are two more equations for the Induction units (Eq. (5)) and Heat exchanger (Eq. (4) and (6)). i represents an order in the iterations from 1 to 1,000.
5) Repeat the process for the whole time-series (j). j represents an order of data points (total data points in the data set: 443,520 points). However, j are different in each category based on the logic which is described below.
6) Calculate mean (${\bar{\dot{Q}}}_{i,j}$) and standard deviation ($\sigma_{{\dot{Q}}_{i,j}}$) of ${\dot{Q}}_{i,j}$ across i=1to1000 at each time step j.

Uncertainties for $\dot{V}$ for the VAVs and induction units were 1 % (T-VER-PX… series) and 4 % (T-DCI-F3xxx series) in Eq. (3), and Eqs. (5) and (6), respectively. In Eq. (4), the reported uncertainty for the Extech AN100 is 3 %, which is needed to calculate ϕ, and $T_{on\;Grille}$ is ± 0.15 °C. Since no uncertainty of $U_{T_{HF}}$ and $U_{BAS}$ has been reported, we assumed uncertainties for both described above. We differentiated the propagation of uncertainty in HVAC energy consumption into heating and cooling energy and also separated the uncertainty of the induction units and heat exchanger due to a relatively complex calculation in the heat exchanger.

**Fig. 16:**
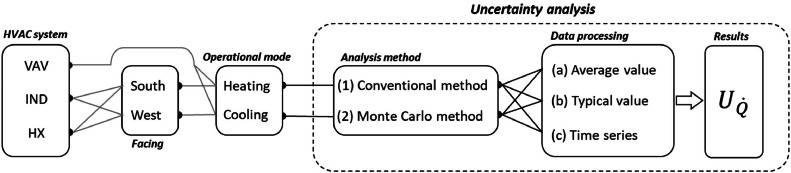
A summary of cases of all uncertainty analyses.

For these two methods (i.e., conventional and Monte Carlo), we applied three different data processing approaches to show different complexities. For the first and simplest approach, a time averaged (or time integrated) approach was used to find the average value for all the data in the study. The second approach finds the median value of the measurements and finds the uncertainty associated with a typical energy value. The third, time-resolved, approach uses the entire time series to find the uncertainty for each time step. A framework of the uncertainty analysis is shown in Figure S6, and Fig. 16 summarizes all cases we explored in uncertainty analysis. The aim is to highlight the similarities and differences that could be beneficial for future studies. Overall, we will have six different methods to calculate uncertainty in the measurements: two methods (i.e., conventional and Monte Carlo) and three approaches (i.e., average value, typical value, and time series). They are represented with the “(method)-(approach)” combination. Here we explored the combinations of each method and approach as: (1)-a, (1)-b, and (1)-c based on the conventional method and (2)-b and (2)-c based on the Monte Carlo method. (2)-a was not pursued as it is simple and does not fit the nature of the Monte Carlo method. Results of uncertainty analysis consist of ten categories of time-series data sets: three mechanical systems with two modes: heating or cooling (six categories overall), and IND and Heat exchanger with two facings: south or west (four categories), two different analysis methods (i.e., the conventional and Monte Carlo) with three data processing approaches. This analysis explored 50 cases of uncertainty in the HVAC system.

## Method validation

This section summarizes a brief overview of the results for the HVAC energy consumption and the propagation of uncertainty. For detailed review of the research findings, please refer to the main research paper [26].

### Scheduling validation

The outdoor condition was the major driver of HVAC system energy consumption. The distribution of outdoor air temperature is a good indicator to determine whether the strategies represent seasonal difference. Table 5 shows the daily average air temperature in the core zone and perimeter zones as well as the daily average outdoor air temperature. Each strategy represents 77 days in the main experiment phase of this study. The indoor air temperature ranged from 21.4 to 22.5 °C, which means the HVAC system controls the indoor air temperature within a 1.1 °C deadband.

**Table 5:** Daily average temperature in the core and perimeter zones as well as the daily average outdoor air temperature for all the strategies.

Temperature ( °C)	Baseline		On-Schedule		Dynamic		Manual	
	Average	Median	Average	Median	Average	Median	Average	Median
Core	22.6	22.7	22.2	22.1	22.5	22.4	22.4	22.5
Perimeter	21.7	21.8	21.4	21.5	21.5	21.7	21.6	22.0
Outdoor	10.5	16.2	11.8	10.4	11.0	12.7	10.4	15.5

**Fig. 17:**
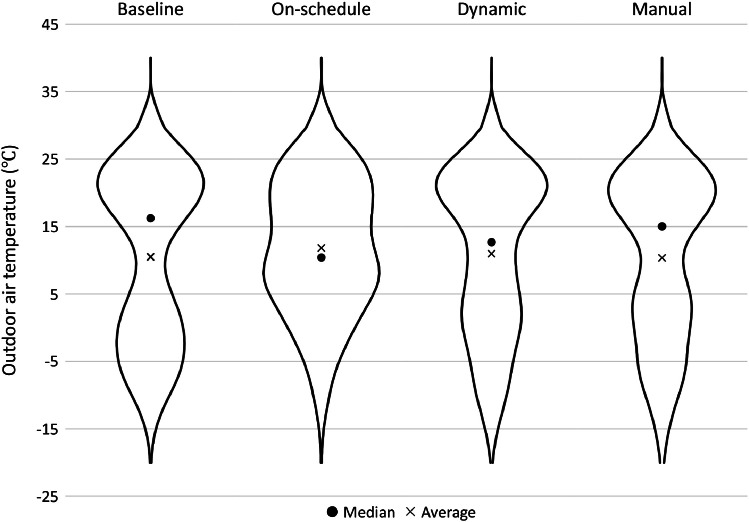
Distributions of daily average outdoor air temperatures during each shade control strategy deployment across the 44-week study from May 2021 to March 2022.

Fig. 17 shows each strategy’s daily average outdoor air temperature profile, including the distribution probability, average, and median for the entire project from May 2021 to March 2022. Distributions of outdoor air temperature during each strategy were not normal (i.e., Shapiro-Wilk test: *p* < 0.05) and there were no significant differences between strategies (i.e., Wilcoxon rank-sum test: *p* > 0.05). Based on these observations, the design of this study was successful in capturing a wide range of seasonally-varying outdoor weather conditions while also randomizing across the four window control strategies.

### HVAC energy consumption

**Fig. 18:**
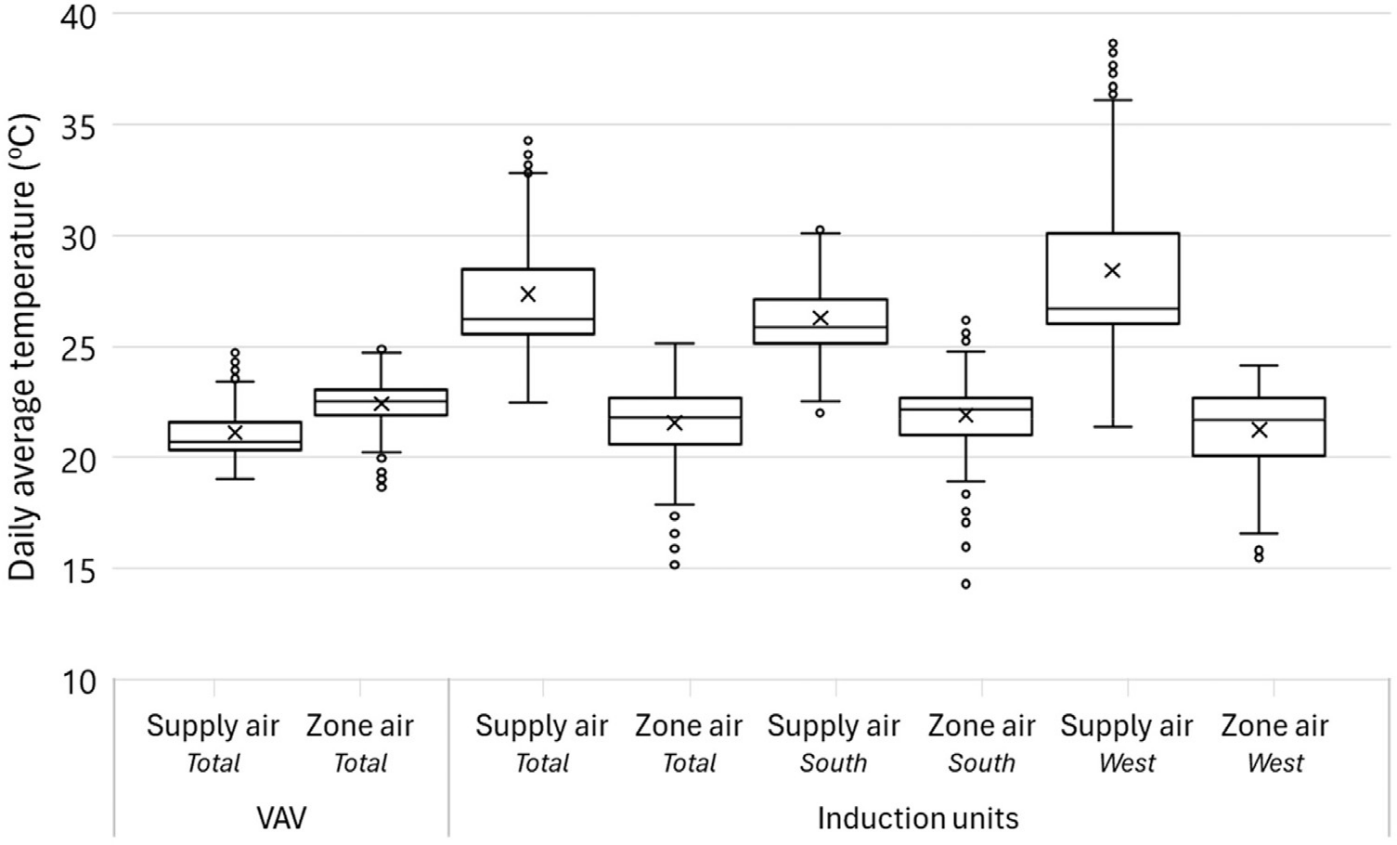
The supply and zone air temperatures across system for different orientations for the entire measurement period from May 3, 2021 to March 6, 2022.

**Fig. 19:**
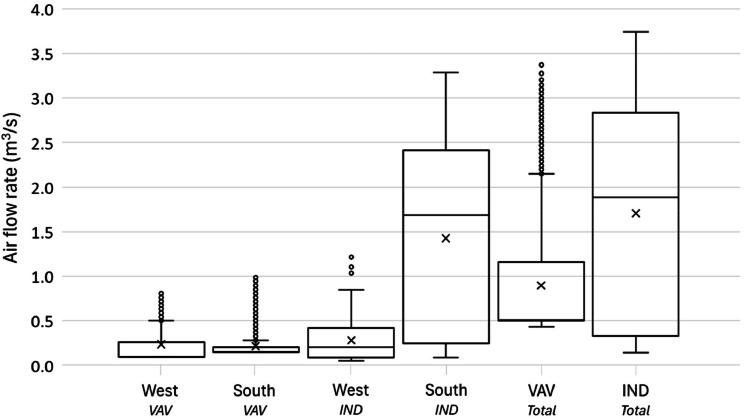
The air flow rate across the system and facing sides for the entire measurement period from May 3, 2021 to March 6, 2022.

Fig. 18 and 19 present the daily average supply and zone air temperatures and air flow rate across the system (e.g., VAV and Induction units) and facing sides (e.g., South and West), respectively. In terms of the total (not split by facing side), the VAV system provided lower temperature (a median of 20.7 °C) than the zone temperature (a median of 22.5 °C), which means its main responsibility is to address the cooling load and only partially contributes to heating load control. The induction units used a higher supply air temperature (a median of 26.2 °C) to control the zone temperature (a median of 21.8 °C). Breaking down the facing side of the daily average temperature for the induction units, the supply air temperature for the west side provided a higher air temperature compared to the south side. Thus, it is concluded that the induction units have different zone air temperature control algorithms depending on the direction which causes the different energy loads.

The air flow rate presented in Fig. 19 is based on 30-minute averaged data. In addition, even if the induction unit data includes all the system in the space because they are located to the perimeter, the VAV system needs to be split into west (VAV‒1, VAV‒5, and a half portion of VAV‒8) and south (VAV‒9, VAV‒10, VAV‒11, a half portion of VAV‒8). In the right-hand side of Fig. 19, the total air flow rate shows a different pattern. The VAV system tightly controls the air flow while the induction units have a wider deadband to provide air. The main portion of induction units’ air flow is noticed in the south-facing units. In this regard, it is concluded that the south-facing induction units control the zone air temperature by providing a high air volume (a median of 1.7 m^3^/s) to the space while the west one supplies a higher temperature difference 5.0 °C while the south one has a gap of 3.7 °C. Standard deviations of the supply air temperature for the total, south, and west are 2.57, 1.76, and 3.65 °C. It indicates that the west-facing induction unit’s supply air temperature relatively has a larger temperature range to control the zone temperature.

**Fig. 20:**
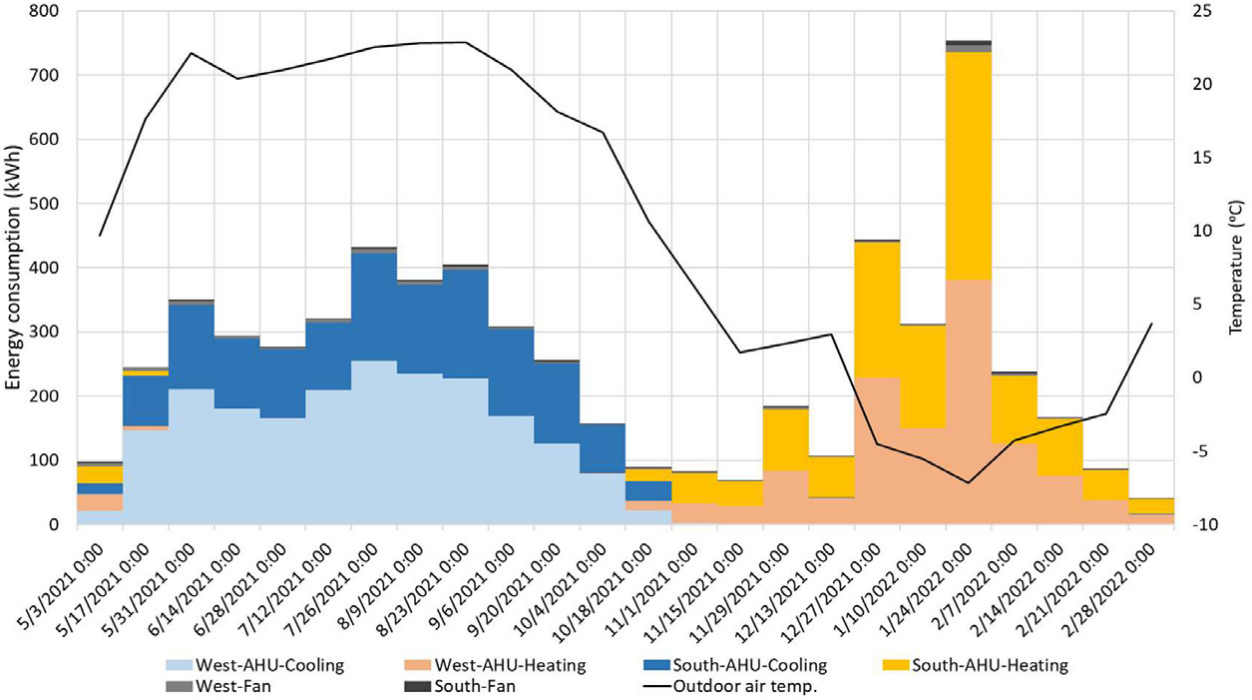
The energy consumption patterns in the VAV system over the entire measurement period from May 3, 2021 to March 6, 2022.

**Fig. 21:**
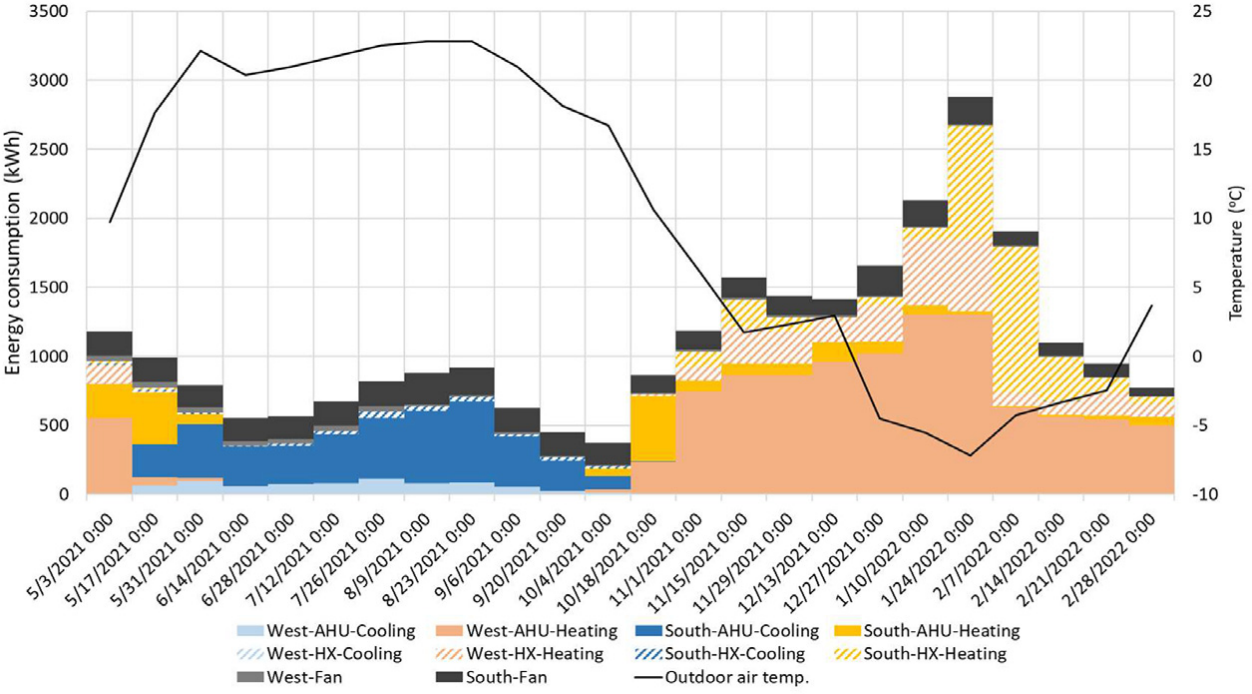
The energy consumption patterns in the induction units energy consumption over the entire measurement duration from May 3, 2021 to March 6, 2022.

This section provides a brief review of the weekly HVAC energy consumption which is based on Eq. (1) and (2). The results of the VAV system and the induction units are shown in Fig. 20 and 21, respectively. It is highlighted that the VAV energy consumption also follows the same condition as the airflow rates in Fig. 19. In other words, the VAV data does not consider the core area shown in Fig. 1. The VAV system consumed more energy to meet the cooling loads compared to the heating loads as shown in Fig. 20. However, it does not mean that there is no contribution to the heating load from the VAV system because, during the peak winter week (1/24/2022 – 2/6/2022), the peak consumption (∼736 kWh) is observed and the VAV system gradually contributed to heating energy consumption.

Fig. 21 shows that the induction unit system has a similar energy consumption pattern to the VAV system. The main difference is that the induction units contributed more to satisfying the heating loads while the VAV system contributed more to satisfying the cooling loads. During wintertime, when the outdoor air temperature decreased, the heating energy consumption increased. In terms of magnitude of energy consumption patterns, the induction units required more energy to adjust the zone air temperature compared to the VAVs (i.e., total energy consumption of VAVs including all VAVs and the induction units in the first phase of study are 12,731 and 26,679 kWh, respectively). Moreover, the fan energy consumption is higher than the VAV, especially for the south-facing induction units leads. When the south-facing side requires more cooling energy, the west-facing units demand is less. Overall, the west requires more heating energy than the south-facing side in the heating season.

### Propagation of uncertainty in energy consumption calculations

While the theoretically available maximum data points from this study are 443,520 for the 10-month measurement duration with 1-min interval data, we counted effective data by applying logical criteria for data inclusion, as described in the Supplementary Material.

**Fig. 22:**
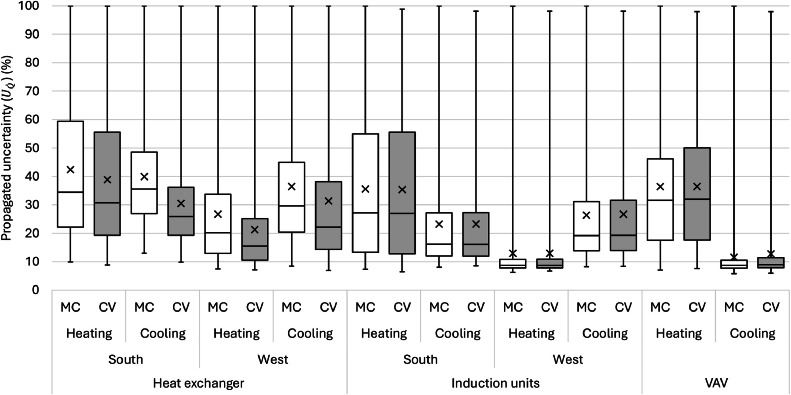
Comparison of time-series uncertainty ($U_{\dot{Q}}$): Conventional (1)-c vs. Monte Carlo (2)-c.

The conventional and Monte Carlo methods were implemented for the time-series data set, and the results of (1)-c and (2)-c are summarized in Fig. 22. From this analysis, the box plot shows how the uncertainty ranged from around 10 to 100 % of uncertainty instead of showing the propagation process, as shown in Figure S8. The bottom and upper bars represent minimum and maximum values. The range of the box shows 25 % to 75 % including median value with the bar and average value with the cross mark. The highest uncertainty was observed in the south-facing heat exchanger worked for the heating followed by VAV for heating, and the lowest one can be found in the west-facing induction units for heating, which is followed by the VAV for cooling. Differences due to the control responsibility and calculation process are presented in Supplementary Material with analysis on (1)-a.

From the left-hand side in Fig. 22, differences in the median values (i.e., subtract Monte Carlo to conventional) are 3.7 %, 9.7 %, 4.7 %, and 7.5 % for the heat exchanger, 0.2 %, 0.0 %, 0.0 %, and ‒0.1 % for the induction units, and ‒0.3 % and ‒0.2 % for the VAV. The differences in average are as follows: 3.5 %, 9.4 %, 5.4 %, and 5.1 % for the heat exchanger, 0.2 %, 0.0 %, 0.0 %, and ‒0.3 % for the induction units, and 0.0 % and ‒1.2 % for the VAV. It is determined that the uncertainties based on the Monte Carlo method for the heat exchanger have higher differences in median and average values compared to other categories, while the Monte Carlo results for the VAV show lower uncertainties than the results based on the conventional method. Regarding the differences in the heat exchanger using two equations (i.e., Eq. (4) and (6)), we also observed the same pattern, described in Supplementary Material based on (1)-a. The conventional method tends to estimate the uncertainty of $U_{\dot{Q}}$ lower than the Monte Carlo. The conventional method with multiplication and division relationship in correlated inputs (called a non-linear function in [93]) results in a systematic shift and its distribution can be asymmetric and flattened [93]. This behavior we observed in the Heat exchanger and the VAV Heating when it is not on the main duty. The induction units having a mid-level complexity with Eq. (5) do not have significant differences between two methods. The Monte Carlo method for the VAV shows slightly lower values than the conventional method. The difference between the VAV and Induction units is that Eq. (5) has one more parameter than Eq. (3) for the VAV. Therefore, when the final uncertainty is made of complex equations, researchers could consider the Monte Carlo method rather than using the conventional one because the Monte Carlo method takes account for the complexity of a propagating process.

Another consideration is that the Monte Carlo method requires more computational effort than the conventional method. To calculate the uncertainty for south-facing Heat exchanger, overall running time with a python code in the Visual Studio Code environment was 127 min. The simulation time for the VAV with the Monte Carlo is around 50 min. The computer set-up is a central processing unit (CPU) of Intel® Core™ i7–9700 CPU @ 3.00 GHz, a solid-state drive (SSD) of Samsung SSD 970 EVO 500GB, and a random access memory (RAM) of 16 GB. It is noted that this study focuses on sharing the methodology and study design, and thus, for additional analysis on the Monte Carlo vs. the conventional, we suggest reviewing the research paper about comparison of two methods [93].

**Fig. 23:**
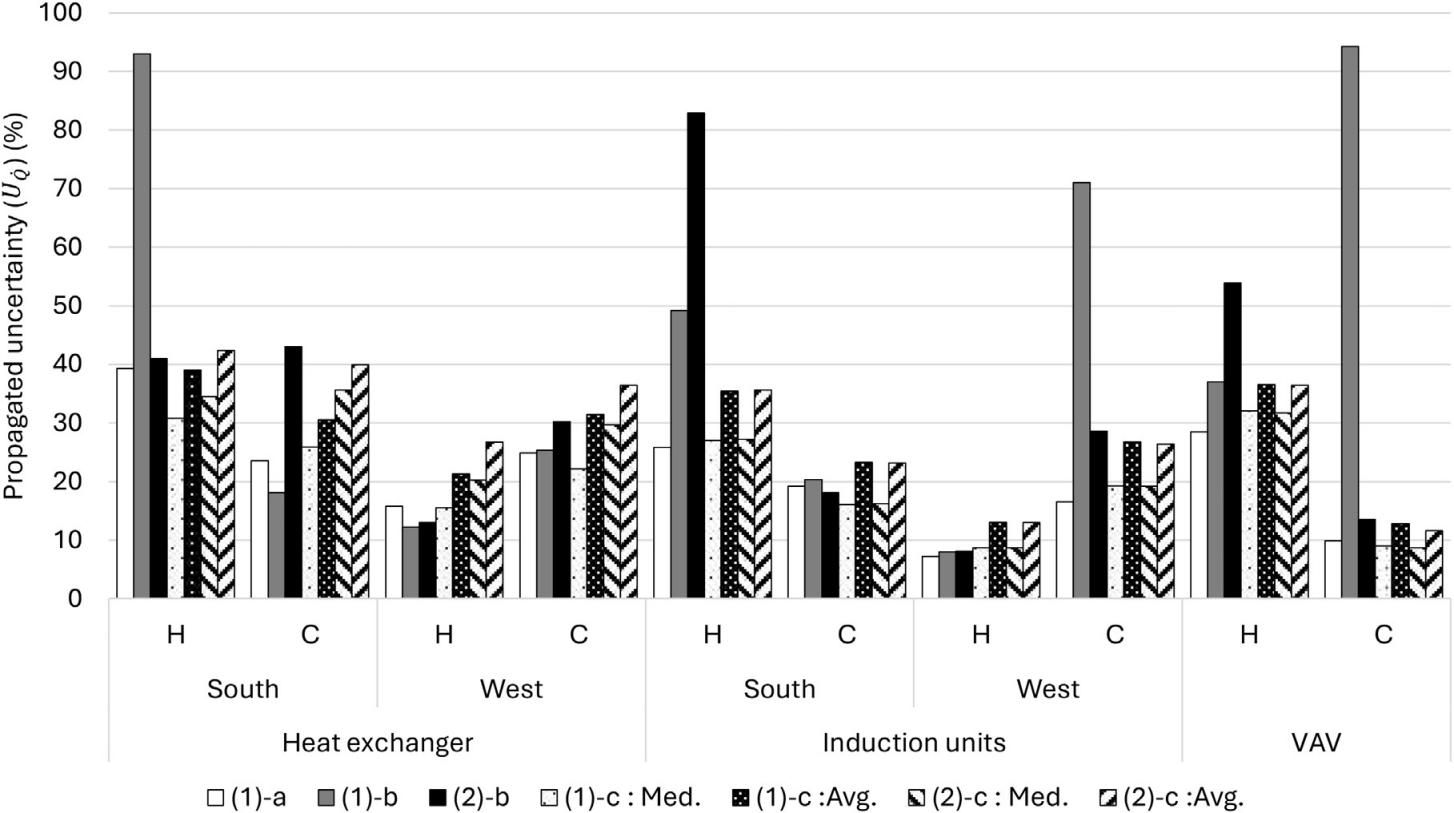
A comprehensive summary of the uncertainties ($U_{\dot{Q}}$) for all the propagation methods and averaging approaches.

Lastly, the uncertainty results using the conventional and Monte Carlo methods are summarized in Fig. 23: (1)-a, -b and -c, and (2)-b and -c. Time dependent uncertainties, (1)-c and (2)-c, show the median and average values. Two analyses, which are based on the median of $\dot{Q}$ and expressed as (1)-b and (2)-b. The high fluctuations than other uncertainties are also shown in Fig. 23. (2)-b shows an off-pattern in the south-facing heat exchanger for cooling (43.0 %), the south-facing induction units for heating (82.9 %), and the VAV for heating (53.9 %). Results in (1)-b tend to exaggerate the uncertainties when it does not have the main responsibility (e.g., 93.0 % and 49.2 % for the south-facing heat exchanger and Induction units and 71.0 % for the west-facing induction units for cooling). In addition, (1)-b shows 94.3 % of uncertainty in the VAV for cooling, which is the highest uncertainty while other analyses (1)-a, (1)-c and (2)-c show low uncertainty (from 8.7 to 12.8 %). It is determined that two analyses based on the Typical value (series of “b”) show a biased representativeness while (1)-a shows a good agreement with the time-series results (series of “c”). The averaging process was conducted across the effective data set, and it could attenuate fluctuations by converging the uncertainty. However, it requires a user’s careful attention in decision-making to take a data processing approach because there would be limited results from our study. Regardless of this limitation, it is still significant when a user, having limited time and computational resources, can use the approach with the averaged values across time, which possibly avoids providing too many biased parameters. For comprehensive understanding, time-series analysis (series of “c”) is highly recommended, and it provides an overall distribution of the uncertainty. In addition, using the Monte Carlo method is recommended because it reflects the complexity more than the conventional method. Thus, a follow-up study trying to adopt the process should review the effectiveness of each method and report uncertainty.

## Limitations

One of the most difficult parts in this study was the QA/QC given the large size of the measured data sets. It is important to maintain the quality of data by minimizing unintentional data loss. A daily review of the data transmission and data quality during the data collection is ideal. This could be achieved by developing a scripted code to call and check the data transmitted and identify major outliers or sensor connections immediately. This study was designed to iterate that each strategy lasted for 2 weeks, and the data was examined at least once a week. The strategies were: (1) On-schedule control that uses a predefined schedule to control the motorized shades, (2) Dynamic (‘smart’) control that takes into account the outdoor condition and indoor occupancy to control the shade positions, (3) Manual control, which utilizes the motorized shades without any control, and (4) Baseline, which is the original mini-blinds when the shades are disabled.

It is highly recommended that additional sensors are deployed to determine the quality of the data on an interim basis. Since they are temporarily installed, a local data storage can be used to secure the data. Relying on one source of data could be detrimental since the sensors might get out of calibration and the quality of data could be questionable. This study used three cloud platforms, and all three of them needed continuous internet Wi-Fi connection to send the data. One platform was able to send the stored data after the Wi-Fi was established. One platform had a built-in SD card that stores the data but does not send the data after the Wi-Fi is re-connected. One of the platforms did not have the storage capability and it was connected to a hotspot.

It is important to note that several other experimental design strategies were also explored but ultimately not pursued. First, ideally, any case-control experiment would be randomized and blinded. However, since the study is in an actual building, randomization of the control strategies would disrupt the daily operation of occupants. Moreover, throughout this study, occupants needed to be informed about each control strategy, and thus we left notes about the operation of the shades at the beginning of each two-week strategy. In addition, we considered different duration for each strategy such as a one week or a day, which are both less than a two-week period, but given the fact occupants had to be involved for almost a year, the two-week duration was selected. This two-week duration allowed capturing performance of each shade in each season. Also, before the start of the study, we assessed utilizing two similar spaces at the building to use one as the baseline (control) and one as the shades (case), but such similar spaces were not available and therefore the design of study was revised to test the shades in the same space.

A differentiating point of this study compared to other studies is how we accounted for the heating and cooling energy end-uses. We especially focused on space conditioning energy analysis because previous studies focused on the lighting energy savings with a side focus on heating and cooling energy end-uses. While originally one goal was to integrate the dynamic control with the light fixtures based on space occupancy, lighting level, time of the day, and outdoor solar irradiance. This goal was not achieved; the integration was more challenging due to several reasons such as lack of interoperability between the BASs (i.e., actual building, window shade, and lighting) or the limitation in installing solar irradiance instruments. Overall, the lighting savings could be insignificant compared to the heating and cooling energy end-uses for cases similar to this building in which the building manager has already installed LED lighting fixtures and occupancy sensors given their short payback periods. The aim of this study was to focus on HVAC heating and cooling energy savings by having the insulated interior shades rather than focusing on the performance of other measures (e.g., an air bubble wrap for windows [94], switchable insulation system for the roof [95], or the insulated sliding panels for shading devices [96]). It is important to note that the methodology described herein can also be extended to other commercial buildings to calculate heating and cooling energy savings of shading interventions. In addition, this study could be used for studies that plan to evaluate the impact of the heating and cooling energy patterns, especially assessing performance of interior shades as an energy efficiency measure.

Another important factor was the close collaboration with the building managers to keep the occupants informed. We needed to coordinate with occupants about the schedules. Without their involvement, this work would have not been feasible for almost a yearlong study. To provide more context, before the start of each two-week strategy, we left a note for occupants in each space on Sundays to inform them about what they would expect in terms of the control strategy for the next two weeks. If the control strategy was On-schedule or Dynamic, more details about the time of shade operations were included in the note for each orientation. In addition, the actual implementation of the strategies required visiting the space at least every two weeks. Before and after each mini-blinds strategy, we needed to consider several practical conditions. We had to go to the office building to adjust the mini-blinds either all the way up or down (i.e., for the Manual control, the mini-blinds had to be all the way up). Also, when there was a mini-blind strategy, the shade switch had to be taken from the space. This means it was confirmed in the real-world setting that the randomized strategies on a lower granularity (i.e., changing every day) was ultimately impractical.

One of the data sources to calculate the HVAC energy consumption was the BAS. The study design was developed independent of the BAS access and during the study it was confirmed that the instrumentation was needed. The BAS could be a good resource for QA/QC but cannot replace the instrumentation. A careful review of the BAS data was needed as the sensors do not go through a continuous QA/QC process. Data obtained from BAS requires either attention to the calibration or at least review of the magnitude of the measurement [97]. This study reported sensing problems to the building engineer, and they replaced the sensor. Finally, it is important to examine the SOO in BAS and confirm the logic of SOO provides the desired output. Some of the SOOs in the BAS for HVAC systems did not match the building operation and they were confirmed in coordination with the building manager.

Last, some occupants were highly engaged with the shades and also challenged the system by overriding the controls or changing the room furniture placements (e.g., adding birthday balloons that blocked the occupancy sensors), which made the actual assessment in our study more realistic, but also more challenging to isolate the impacts of shades and shade controls alone in a complex environment [98].

## CRediT author statement

**Jongki Lee:** Methodology, Software, Validation, Data curation, Writing- Original draft preparation, Visualization, Investigation, Writing- Reviewing and Editing. **Akram Syed Ali:** Conceptualization, Methodology, Software, Validation, Visualization, Writing- Reviewing and Editing. **Saman Haratian:** Methodology, Software, Validation, Visualization, Writing- Reviewing and Editing. **Brent Stephens:** Conceptualization, Methodology, Validation, Investigation, Supervision, Writing- Reviewing and Editing. **Mohammad Heidarinejad:** Conceptualization, Methodology, Software, Validation, Data curation, Writing- Original draft preparation, Visualization, Investigation, Supervision, Writing- Reviewing and Editing.

## Declaration of competing interest

The authors declare the following financial interests/personal relationships which may be considered as potential competing interests: This work was supported by the Emerging Technologies initiative within the ComEd Energy Efficiency Program. Also, this study was also funded in part by an ASHRAE New Investigator Award to Mohammad Heidarinejad.

## Data Availability

Data is unavailable to access or unsuitable to post as the research data includes confidential information. In coordination, part of the data could be shared on reasonable request.

## Appendix Supplementary materials

Image, application 1
