# Supplementary material for: A framework to evaluate the thermal and energy performance of smart building systems in existing buildings: A case study on automated interior insulating window shades
Source: MethodsX. 2025 May 19;14:103378. doi: 10.1016/j.mex.2025.103378 (PMC12221587; doi:10.1016/j.mex.2025.103378)
Supplement: Supplementary file 1 [file mmc1.docx]

# Supplementary material information

**Article title**

A Framework to Evaluate the Thermal and Energy Performance of Smart Building Systems in Existing Buildings: A Case Study on Automated Interior Insulating Window Shades

**Authors**

Jongki Lee, Akram Syed Ali, Saman Haratian, Brent Stephens, Mohammad Heidarinejad*

**Affiliations**

Department of Civil, Architectural, and Environmental Engineering, Illinois Institute of Technology, Chicago, IL 60616, USA

**Corresponding author’s email address and Twitter handle**

muh182@iit.edu

**Keywords**

Energy performance; building science; field measurement and validation; heating ventilation and air conditioning systems; energy efficiency measure

**Specifications table**

| **Subject area** | Engineering |
| --- | --- |
| **More specific subject area** | Measurement and data analysis in an existing building to assess energy performance |
| **Name of your method** | A methodological framework to evaluate the thermal and energy performance of interior insulating window shades in existing buildings |
| **Name and reference of original method** | Jongki Lee, Akram Syed Ali, Afshin Farmarzi, Urwa Irfan, Christopher Riley, Brent Stephens, Mohammad Heidarinejad, Assessing the long-term energy performance of automated interior insulating window shades in a high-rise commercial building, Applied Energy, Volume 378, Part B, 2025, 124797, ISSN 0306-2619, https://doi.org/10.1016/j.apenergy.2024.124797. |
| **Resource availability** | All the instruments and their datasheets are cited in the text. |

This supplementary material file summarizes additional information on (1) data quality assurance and quality control (QA/QC) for the variable air volume (VAV) and induction unit systems and (2) the process to calculate the uncertainty associated with measurements.

# Additional sensors and data quality assurance and quality control

## Variable Air Volume (VAV) boxes

At the zone level, the 11 diffusers connected to the 11 VAV boxes were monitored to measure supply air temperature and relative humidity using Onset MX 1104 data loggers [1]. Figure S1 shows the sensor installed in parallel to the U-12 HOBO loggers [2]. Early on, for a few ceiling diffusers and for a few weeks, the loggers were installed in parallel for QA/QC purposes. The loggers were hidden from the occupants as they were installed immediately after the air supply between above the face cover.


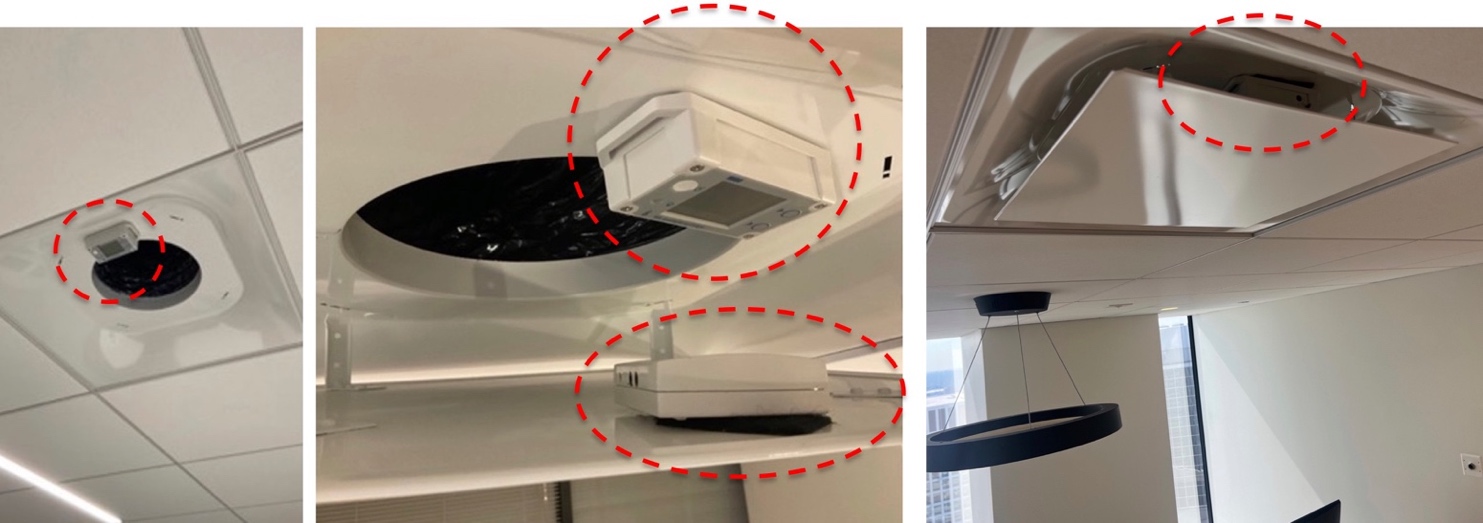


Figure S1. The installation of ceiling diffuser sensors.

There is always a need to conduct continuous QA/QC during the study and, if possible, include additional methods to measure similar variables for redundancy and greater data capture. Since the pressure transducers were powered by batteries, it was expected that the batteries would not last for the duration of the study design, but the exact period of failure was unpredictable. As such, the operation of the pressure transducers was continuously monitored. In August, which is roughly about 10 months after the batteries were initially installed, it was observed that the air flow from the customized boxes showed abnormal patterns. The air flow rate data from the measurement is expressed with a semi-transparent gray color line graph on the top of BAS data represented with a black color line. Figure S2 shows that there was a sudden increase of air flow rate from 8/13/2021 to 9/6/2021, and a decrease in the following period by 10/17/2021. Compared to other time periods, this was an indicator that there is a need to review the data carefully with the other variables. Therefore, the BAS data was used to conduct a detailed QA/QC process.

Figure S2. Total air flow comparison of the values obtained from the BAS and the measurements.

As shown in Figure S3, after checking the battery, we validated our readings from the customized boxes with an Energy Conservatory DG-700 differential pressure gauge [5]. Figure S3(a) shows the battery voltage measurements. In our study, it seems the newer differential pressure transducers, T-VER-PX3UL, benefited from a low-voltage upgrade and they lasted longer that prior experiences [6]. Figure S3(b) and (c) shows when the value of the readings is compared using the DG-700 pressure gauge and the readings from the custom boxes.

| 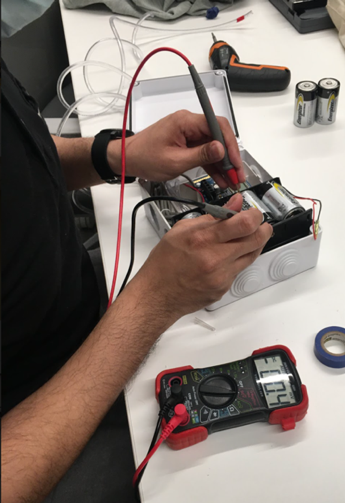  *(a)* | 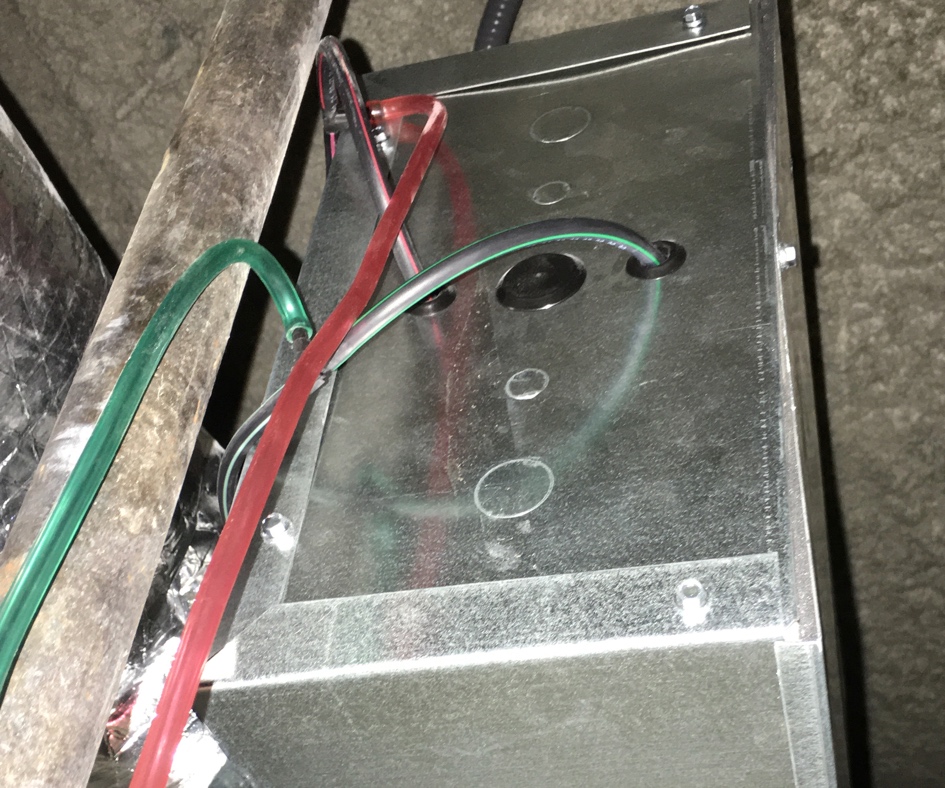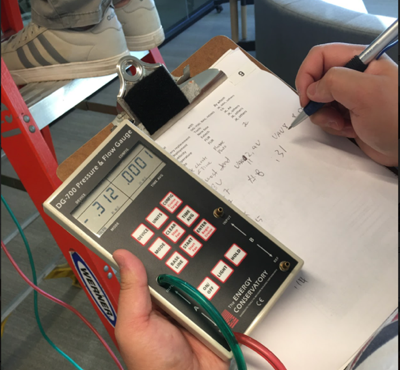  *(b)* | 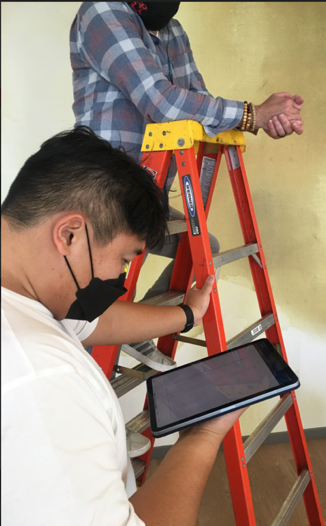  *(c)* |
| --- | --- | --- |

Figure S3. The QA/QC process for the customized differential pressure box on October 18, 2021.

The comparison indicated that the sensors began to measure the airflow rates incorrectly, suggesting the need to replace the batteries inside of the customized pressure transducer boxes. Figure S3 shows the process of checking the batteries during and after the replacement. After the batteries were replaced, the measured data were used. However, there was a need to ensure the same data resolution and accuracy was used for the duration of the abnormal period as shown in Figure S2. A calibration process was done with the values from BAS and the measured data using a linear regression. Figure S2 shows that there is a baseline air flow rate for a case when BAS air flow rate is lower than 1000 m^3^/h which seems to a drift over time, indicating that in the linear regression there is a fixed baseline. Figure S4 shows three components in the air flow comparison between the measurement and BAS data: (i) correlated data points expressed by circles; (ii) uncorrelated data point when BAS is under 1000 m^3^/h expressed by triangles, and (iii) a fixed baseline air flow rate for a case when BAS air flow rate is lower than 1000 m^3^/h; and developed linear regression line based on two variables (Measurement and BAS). Figure S4(a) shows that before the observation of an abnormal pattern in the air pressure transducers corresponding to a left hand-side of Figure S2 before the yellow dashed boxes (5/3/2021 to 8/12/2021). First, a calibration curve was developed using the linear regression between the measurement and BAS data as shown in Figure S4(a). Before the event shown in Figure S4(a), this period showed a good coefficient of determination (R^2^) with air flow rate data sets from the BAS and measurement with a baseline of 1813.5 m^3^/h. After QA/QC, the customized boxes shown Figure S4(b) were able to have a similar R^2^ value (0.98) and 1808.5 m^3^/h baseline air flow rate. Furthermore, data from the period having the abnormal pattern shown in the yellow-dashed boxed in Figure S2 was replaced with data regenerated using the correlation with the BAS data. Overall, this process and the extra data collection empowered to utilize the data as needed to ensure there is no data loss during the field. While it is very common to experience field data loss, this study was able to minimize it to zero with the described QA/QC approaches.

Figure S4. The air flow rate comparison between measurement and BAS: (a) from 5/3/2021 to 8/12/2021 and (b) from 10/19/2021 to 10/25/2021 after the battery replacement.

## Induction units

The next system that also benefited from QA/QC with additional sensor installation is the induction unit system. We deployed 12 more sensors (six for air velocity: DIGI-SENSE 20250-22 [3], and six for temperature and relative humidity: HOBO U12 [2]), as shown in Figure S5. The anemometer and temperature sensor were paired and evenly distributed per each side. These sensors were prepared to provide back-up data to ensure that both sides had the same values and to account for any potential losses in Wi-Fi connection in the custom box. Furthermore, the set-up required on-site downloading, which we attempted to do within approximately 3-week intervals to regularly check on the equipment and ensure data were collected successfully. To calculate the contribution of the heat exchanger related to the induced air, we measured the air flow rate by using the Extech AN100 [4].

| 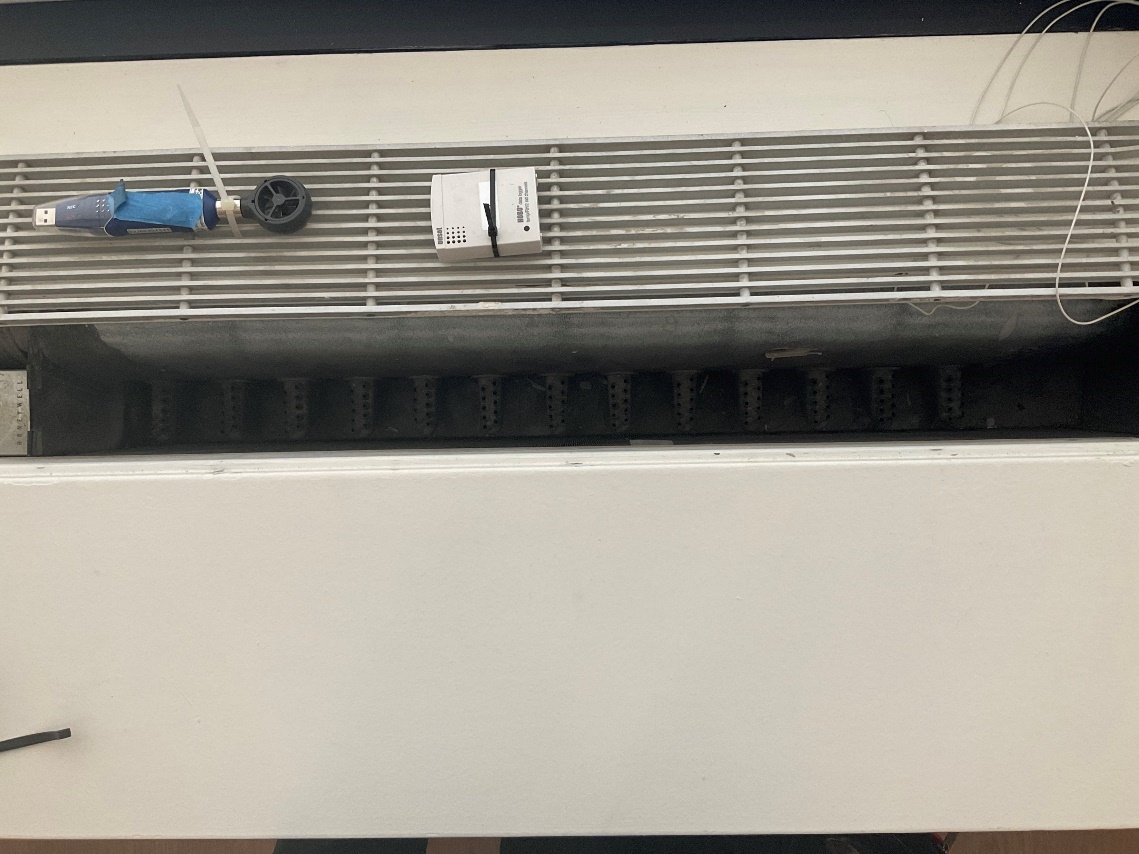 | 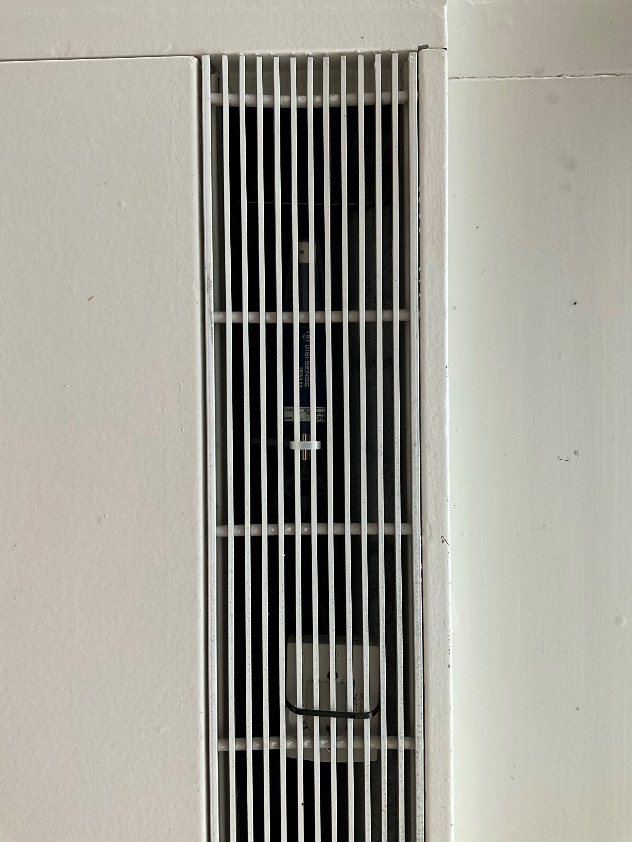 |
| --- | --- |
| *(a)* | *(b)* |

Figure S5. Installation of anemometer and temperature and relative humidity sensor in the grille of induction units: (a) grille off and (b) grille on.

# Calculating the uncertainty associated with measurements

The details of the three approaches to uncertainty estimation are as follow:

1. Calculate the uncertainty of $U_{\dot{Q}}$ based on average $T_{after\_coil}$ and $T_{before\_coil}$ across the whole experiment period, called the “Average value” method. The process is to find the average temperatures and calculate the uncertainty ($U_{\dot{Q}_{Temp,Avg}}$) following *dashed lines* from 1 to 2 in Figure S6.
2. Calculate the uncertainty of $U_{\dot{Q}}$ based on parameters $T_{after\_coil}$ and $T_{before\_coil}$ when $\dot{Q}_{Median}$ is observed, called “Typical value”. The process is to find the median of $\dot{Q}$ in the data set and use temperatures resulting $\dot{Q}_{Median}$ to calculate the uncertainty ($U_{\dot{Q}_{Median}}$) following *dotted lines* from 1 to 3 in Figure S6.
3. Calculate the uncertainty of $U_{\dot{Q}}$ in each time point and summarize all $U_{\dot{Q}}$ across the whole experiment period, expressed as $U_{\dot{Q}_{[0,i]}}$. The process is following *solid lines* from 1 to 2 in Figure S6 and called “Time series”.

Figure S6. The proposed framework to conduct the uncertainty analysis: “a” with the dashed lines is for the Average value approach, “b” with dotted lines is for the Typical value approach, and “c” with solid lines is for the Time series approach.

Figure S7 shows the data cleaning process. While the maximum possible total number of data points for a variable could be 443,520, which corresponds to the field measurement spanning over 10 months (~310 days) at 1-minute intervals (i.e., 60 data points per hour multiplied by 24 hours per day multiplied by around 310 days). We applied the following logic to clean the data and find the effective data. Within the process we applied three data cleaning steps to exclude problematic data: (i) consider the operational mode (i.e., summer or winter) that means the data is less than the maximum total value for almost all the variables as we either calculate heating or cooling, (ii) consider the HVAC working hours meaning when the system is on (e.g., mostly 6 am to 5 pm) which will remove the zero energy data, and (iii) consider only relative uncertainty that is less than 100%, as a small value could lead to unrealistic uncertainty while the actual uncertainty is insignificant. The order of implementation is as follows from step (i) to step (iii) sequentially. The number of data points we used to calculate the uncertainty for $U_{\dot{Q}}$ shows in the Y-axis and the secondary Y-axis shows the percentage of the data numbers compared to the total data points.

The first step which is the Operational mode aims at understanding the summer or winter conditions based the relationship between two temperature points with the operational mode. The logic for the Operational mode is as follow:

- VAV: $T_{MA}>T_{DA}$ and “summer” mode OR
   $T_{MA}<T_{DA}$ and “winter” mode
- IND: $T_{MA}>T_{DA}$ and “summer” mode OR
   $T_{RA}>T_{DA}$ and “winter” mode

Heat exchangers in the IND: $T_{Zone}>T_{HX}$ and “summer” mode OR
 $T_{Zone}<T_{HX}$ and “winter” mode

Then, in the second step, the HVAC working hours is considered. This corresponds to when the HVAC systems are actively turned on and in operation with no setback temperature in place to control the room temperature. Furthermore, through our research on HVAC systems, the VAV and induction units including the heat exchangers at the zone level have a floating mode that refers to the HVAC system and does not actively control the room temperature. Thresholds for determining whether the HVAC systems actively control the temperature or not are based on the 25% percentile of the air flow rate. It is noted that data points under 25% percentile of the air flow rate in the measurement period were excluded. This is specific to this building as the SOOs for the HVAC systems are usually based on 7 am to 6 pm.

In the third step, only uncertainty values equal to or below 100% were considered and the data points having uncertainty over 100% were excluded. We meticulously reviewed data points showing over 100% of the uncertainty, and the common point of these points are having extremely small temperature differences across the coil (e.g., 0.0108 ℃, 0.05 ℃, 0.098 ℃, 0.055 ℃, and 0.022 ℃, etc.), which are functionally not indicative of active operation. The overall pattern is a larger number of data points after processing is observed when the HVAC system has the main responsibility as shown Figure S7. However, it is noted that the induction units for the south-facing side showed more data points for cooling. The heating remains in terms of the main responsibility in the induction units because this analysis is not able to describe the magnitude of energy transfer instead of counting the data points.


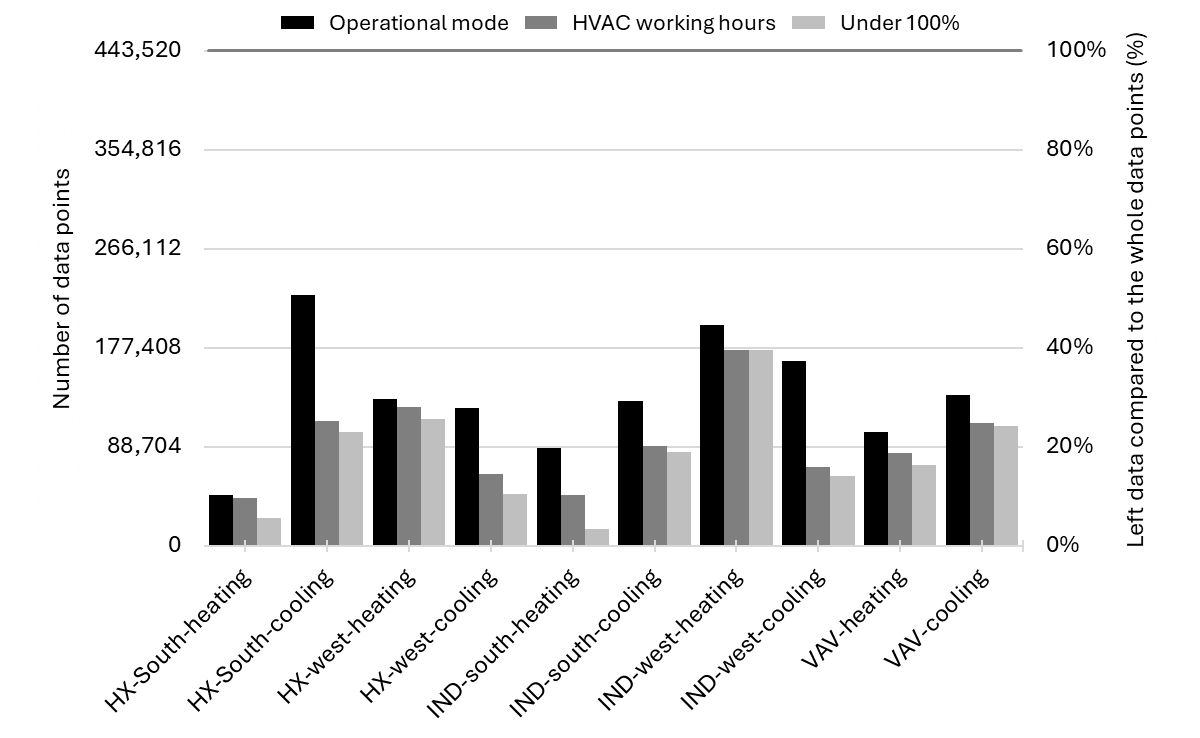


Figure S7. Number data points remaining for analysis after data processing in the Time dependent approach with the Monte Carlo method, (2)-c.

This study included two methods to propagate uncertainty associated with the time-series data from the HVAC systems: (1) the conventional method and (2) the Monte Carlo method. Based on different methods and the approaches, the results are presented as follows:

- (1)-a: The Average value approach using the conventional method (shown in Figure S8 and Figure 23)
- (1)-b: The Typical value approach using the conventional method (shown in Figure 23)
- (2)-b: The Typical value approach using the Monte Carlo method (shown in Figure 23)
- (1)-c: The Time Series approach using the conventional method (shown in Figure 22 and Figure 23)
- (2)-c: The Time Series approach using the Monte Carlo method (shown in Figure 22 and Figure 23)

This means for the Time Series approach the uncertainty values can be plotted as a box plot figure while for others there is a single value. Consequently, for the Typical (approach “b”) and Average (approach “a”) only one instantaneous value can be shown.

Figure S8 summarizes the results from the Average value approach with the conventional method, (1)-a, which can be easily accessible for researchers and building engineers. The heating energy consumption for the VAV system has a much higher uncertainty than the cooling energy consumption for the VAV system (i.e., 28.5% and 9.9%, respectively). In our main research paper and according to analysis for Figure 18, it was concluded that the main responsibility of the VAV system is to meet the cooling energy loads. Regarding this, it is confirmed that a small temperature difference in the heating season causes a higher uncertainty compared to the cooling season when the temperature difference has a higher temperature gap to handle the cooling loads [7]. The temperature differences for the cooling and heating modes are 7.5 ℃ and 2.5 ℃, respectively. The same patterns were observed in the induction unit system in the zone level (called the heat exchanger) and AHU level for the west-facing units because to adjust the indoor air temperature it delivers a higher temperature gap compared to the south one. The temperature gaps for the west and south facing units are 10.5 ℃ and 4.0 ℃ for the heat exchanger for cooling and 5.6 ℃ and 6.3 ℃ for heating, respectively. The AHU level also shows the same pattern as the heat exchanger. The temperature gaps for the west and south facing units show 4.4 ℃ and 3.8 ℃ for cooling and 11.8 ℃ and 2.8 ℃ for heating, respectively. It is noted that the south-facing units show higher uncertainty in the heating season since its mechanism to control the indoor air temperature is opposite to the west-facing units (i.e., high air volume to the space instead of high temperature gap).

Based on the results from Figure 18, Figure 19 and Figure S8, it is obvious that the operational mechanism (e.g., how to control the room temperature) is the one of drivers of increasing uncertainty. According to Equation (14), a simple form of uncertainty calculation for this study, we observed the reported numeric error highly depending on the measured value compared to the fractional error, which is consistent in the equation. The uncertainty of the south- and west-facing heat exchangers shows higher uncertainties compared to the AHU’s uncertainties. The intrinsic complexity in the heat exchanger (Equation (4) and (6)) caused high uncertainty. Comparing the main load responsibilities for the VAV and Induction units (west only) from the AHU level, their uncertainties are 9.9% and 7.2%, respectively. However, the heat exchanger shows 15.8%, which is relatively higher than other results. The south facing Induction units showed higher uncertainty compared to the west facing one because it has a different mechanism to control the indoor temperature (i.e., more air volume and less temperature gap compared to the west facing one). In this regard, the first thing a researcher who is seeking uncertainty from their system should do is review intrinsic complexity with an equation they use to determine what parameter drives the uncertainty in the system.

The heat exchanger is a good example of how uncertainty propagates in the system. For example, referring to Equation (4), the first bar from left to right is $U_{\phi}$, and it is merged with $U_{T_{HF}}$ to calculate $U_{\phi T_{HF}}$. By using Equation (12), $T_{on Grille}-T_{HF}+\phi_{Side}T_{HF}$ is calculated. $U_{T_{HX}}$ needs to use Equation (13) with the result from the previous process. Results of (1)-a are summarized in Figure S8. The first two columns are similar across the four categories in the heat exchanger division. From the third column, the west facing heat exchanger has higher uncertainty compared to the south one. However, the temperature gap significantly impacts on the final uncertainty because even if the third column for the south facing heat exchanger for the heating shows 8.8%, it goes up to 38.8% at the fourth column. A disadvantage of using the conventional method is related to the calculation process because a user needs to formulate equation(s) for uncertainty.

Figure S8. The propagation of uncertainty ($U_{\dot{Q}}$): Average value approach with the conventional method, (1)-a.

# Acknowledgments

The authors would like to thank the ComEd team: Steven LaBarge and Jared Slucter; CLEAResult: Tim Cycyota; Parata team: Christopher Nurre and Dick Co; the Amatis/Nextek team: Sebastien Gouin-Davis, Jeff Daudert, Chris Radke, Karl Heusinkveld, and Nicolas Theoret; Somfy Systems: David Townslee; Lafayette Interior Fashions: Joe Nash Morgan III, Drew Schubert and Derek Bolhoffer, and IIT team: Afshin Faramarzi, Christopher Riley, Urwa Irfan, Rémi Thelier, and Brianna Galvan. Finally, the authors would like to acknowledge the close collaboration of the Equity Office management and staff at Willis Tower for their patience during this project. They have graciously supported this project and all the visits. We appreciate the full support of the Equity Office management and staff.

# References

[1] Onset, HOBO® MX1104 Data Logger: Analog/Temp/RH/Light, (2019). https://www.onsetcomp.com/products/data-loggers/mx1104 (accessed June 19, 2024).

[2] Onset, Onset, HOBO U12 Temp/RH/Light/External Data Logger, (2022). https://www.onsetcomp.com/files/manual_pdfs/13128-C%20U12-012%20Manual.pdf.

[3] Digi-Sense, Data Logging Anemometer: Model 20250-22, (2022). https://pim-resources.coleparmer.com/instruction-manual/digi-sense-20250-22-data-logging-anemometer-instruction-manual.pdf.

[4] FLIR, Extech AN100: CFM/CMM Mini Thermo-Anemometer, (2024). https://www.flir.com/products/an100?vertical=condition+monitoring&segment=solutions (accessed July 1, 2024).

[5] The Energy Conservatory, DG-700 Pressure and Flow Gauge, The Energy Conservatory | Blower Door | Duct Blaster (2012). https://energyconservatory.com/support/dg-700-user-manual/ (accessed July 1, 2024).

[6] T. Ramos, S. Dedesko, J.A. Siegel, J.A. Gilbert, B. Stephens, Spatial and Temporal Variations in Indoor Environmental Conditions, Human Occupancy, and Operational Characteristics in a New Hospital Building, PLoS ONE 10 (2015) e0118207. https://doi.org/10.1371/journal.pone.0118207.

[7] D.S. Borges, R.D. Hermans, D.B. Bivens, P.W. Cabot, H.F. Crowther, B.P. Dougherty, H. Elmahdy, M.R. Hargan, R.L. Hedrick, J.F. Hogan, F.E. Jakob, S.D. Kennedy, D.E. Knebel, M.F. McBride, M.P. Modera, C.H. Nasseri, D. Novosel, G. Reeves, J. Sabelli, S.V. Santoro, G. Shavit, D.R. Tree, J.E. Woods, M.F. Beda, W.A. Harrison, C.B. Ramspeck, ASHRAE Guideline 2-2005: Engineering Analysis of Experimental Data, (2005).
